# Network Pollution Games

**DOI:** 10.1007/s00453-018-0435-4

**Published:** 2018-04-02

**Authors:** Eleftherios Anastasiadis, Xiaotie Deng, Piotr Krysta, Minming Li, Han Qiao, Jinshan Zhang

**Affiliations:** 1 University of Liverpool, Liverpool, UK; 2 Shanghai Jiao Tong University, Shanghai, China; 3 City University of Hong Kong, Hong Kong, China; 4 University of Chinese Academy of Sciences, Beijing, China

**Keywords:** Algorithmic mechanism design, Approximation algorithms, Planar graphs, Pollution control

## Abstract

The problem of pollution control has been mainly studied in the environmental economics literature where the methodology of game theory is applied for the pollution control. To the best of our knowledge this is the first time this problem is studied from the computational point of view. We introduce a new network model for pollution control and present two applications of this model. On a high level, our model comprises a graph whose nodes represent the agents, which can be thought of as the sources of pollution in the network. The edges between agents represent the effect of spread of pollution. The government who is the regulator, is responsible for the maximization of the social welfare and sets bounds on the levels of emitted pollution in both local areas as well as globally in the whole network. We first prove that the above optimization problem is NP-hard even on some special cases of graphs such as trees. We then turn our attention on the classes of trees and planar graphs which model realistic scenarios of the emitted pollution in water and air, respectively. We derive approximation algorithms for these two kinds of networks and provide deterministic truthful and truthful in expectation mechanisms. In some settings of the problem that we study, we achieve the best possible approximation results under standard complexity theoretic assumptions. Our approximation algorithm on planar graphs is obtained by a novel decomposition technique to deal with constraints on vertices. We note that no known planar decomposition techniques can be used here and our technique can be of independent interest. For trees we design a two level dynamic programming approach to obtain an FPTAS. This approach is crucial to deal with the global pollution quota constraint. It uses a special multiple choice, multi-dimensional knapsack problem where coefficients of all constraints except one are bounded by a polynomial of the input size. We furthermore derive truthful in expectation mechanisms on general networks with bounded degree.

## Introduction

The advance of technology and commercial freedom have fused and accelerated the development process in an unprecedented scale. Environmental degradation however has accompanied this progress, resulting in global water and air pollution. In many developing countries, this has caused wide public concerns. As an example, in 2012, China discharged 68.5 billion tons of industrial wastewater and the SO$_2$ emissions reached 21.2 million tons (National Bureau of Statistics of China, 2013). China has become one of the most polluted countries in the world with industrial emissions as the main source of its pollution. The recent annual State of the Air report of the American Lung Association finds that $47\%$ of the Americans live in counties with frequently unhealthy levels of either ozone or particulate pollution [4]. The latest assessment of air quality, by the European Environment Agency, finds that around $90\%$ of city inhabitants in the European Union are exposed to one of the most damaging air pollutants at harmful levels [1]. Environmental research suggests that water pollution is one of the very significant factors affecting water security worldwide [57].

It is the role of regulatory authorities to make efficient environmental policies in balancing economic growth and environment protection. Pollution control regulations are inspired by the managerial approaches in environment policies, where models based on game theory are proposed and analysed. Kwerel [38] proposed a mechanism where firms, potential polluters, report their clean-up cost information to the regulator. The regulator sells a fixed number of pollution licences at a fixed price per licence and offers a subsidy for those licences which firms hold in excess of emission, based on the cost information provided by firms. In Kwerel’s mechanism truth-telling by all firms implies a Nash equilibrium. Kwerel’s scheme maintains a mild level of pollution by optimizing the social welfare (sum of the global clean-up cost and damage cost of emitted pollution).

From a different point of view, Dasgupta et al. [15] focus on minimizing the sum of pollution damages, abatement costs and individual rationality for consumers. Spulber [50] develops a market model of environmental regulation with interdependent production, pollution abatement costs and heterogeneous firms who have private information about costs and pursue Bayes-Nash strategies in communication with the regulator. Their paper illustrates that the full information optimum cannot be attained unless gains from trade in the product market net of external damages exceed the information rents earned by firms and aggregate output and externality levels are lower at the regulated equilibrium than at the full information social optimum.

In a given geographic area, there are owners of pollution sources (e.g., factories, cars). The owners of these sources are interested in buying licences, i.e., permits, for the emission of pollution. The government, as a regulator, is responsible for allocating the licences to the owners in such a way that the amount of emissions does not exceed certain levels both globally and locally, i.e., the regions around the pollution sources. This allocation aims at the maximization of owners’ satisfaction, that is, the social welfare is maximized. See Sect. 3 for details.

Pollution has a diffusion nature: emitted from one source, it will have an effect on its neighbours at some decreased level. We consider two applications using a network model. In the first application, the vertices represent pollution sources and edges are routes of pollution transition from one source to another, similar to Belitskaya [11]. Our model measures the pollution diminishing transition by arbitrary weights on the edges, which are also present in the model of Montgomery [41]. The polluters’ privately known clean-up cost and damage of the emitted pollution in our model are inspired by Kwerel [38]. In the second application, the vertices represent mayors of cities and the edges represent the roads between cities. The percentage of cars moving from one city to another is represented by the weight of the corresponding edge. Note, that in this application, although cars are physical sources of pollution, vertices, i.e., cities to which cars drive, are factual pollution sources. Thus, also in this application, the vertices (cities) can be regarded as pollution sources.

Our model covers both aforementioned applications with details given in Sect.  3. The government, as the regulator, can decide to either shut down or keep open a pollution source taking into account the diffusion nature of pollution. It sets bounds on the global and local levels of pollution, while trying to optimize the social welfare. The emissions that exceed the amount of pollution allowed by the licences, if any, must be cleaned-up, incurring an additional cost to the agent, called agent’s clean-up cost. Furthermore, in the second application of our model, the regulator is allowed to auction pollution licences for cars to mayors. In this case, the pollution level of an agent (mayor), i.e., the number of allocated licences, is set by the regulator together with the prices that the agent pays to get them.

Furthermore we study water pollution in rivers modelled by tree networks. In water pollution the government decides which pollution sources should be shut down so that the effluent level in water is as low as possible. Water pollution cost sharing was introduced in [42] where the network is a path (single river). This model was extended to tree networks (a system of rivers) in [18]. We model a system of rivers as a tree, but study a different pollution control model.

As a variant of the first application described above, we also consider the case in which the government is allowed to sell licences to the pollution sources instead of deciding to shut them down or keep them open. This is a widely used approach to control pollution levels by auctioning a fixed number of licences or pollution allowances. For instance, the European Emissions Trading System sells EU Emission Allowances (EUAs), each one representing the right to emit one ton of CO$_2$. In such an auction, firm’s bid is a number of EUAs and per EUA a price. The auction ranks all the bids in descending order of per EUA price and determines the per EUA clearing price. The clearing price is the first bid price such that the total volume of EUAs in the bids (demand) in this descending order meets the total volume of EUAs offered by the regulator (supply). All the bids above this clearing price are awarded and they all pay the clearing price, see, e.g., [2]. This very simple auction does not take into account the diffusion relations between polluters, etc.

Finding an optimal social welfare solution to our problem, which we call Pollution Game (PG), is NP-hard, that is why we study polynomial time approximation algorithms which can lead to incentive compatible (truthful) mechanisms. We study linear cost and damage functions and derive approximation algorithms and truthful mechanisms focusing on planar network topologies. In contrast, Belitskaya [11] assumes quadratic cost functions and linear damage functions deriving optimal social welfare and Nash equilibria solutions by explicit analytic formulas. We focus our study on planar network topologies which model realistic scenarios.

Most of the cited economics papers derive equilibria by closed analytic formulas. Some of these papers provide computational mechanisms without guaranteeing polynomial running time. Our approach is algorithmic and focuses on efficiently computing these solutions. We also analyze the computational complexity/hardness, of computing the social optimum in our model. To the best of our knowledge, this work is the first attempt to algorithmically analyze pollution control from the perspective of regulators by a network game model with information asymmetry between regulators and polluters.

We mainly study linear objective functions on trees and planar graphs. When the network is a directed tree, a somehow non-standard two level dynamic programming approach is designed to obtain an FPTAS for our pollution game (PG). This approach is crucial to deal with the global pollution quota constraint. It uses a special multiple choice, multi-dimensional knapsack problem where coefficients of all constraints except one are bounded by a polynomial of the input size.

Baker’s shifting and tree-width decomposition techniques, see, e.g., [8, 30], are used for designing PTASs for various problems on planar graphs. It seems unlike to design a PTAS for PG with binary variables on planar graphs by adapting these techniques. That is because they deal with constraints on edges (e.g., for the independent set problem), but PG’s constraints are imposed on vertices from its neighbouring vertices. More precisely, in the independent set problem, the number of nodes is bounded (at most one) per each edge, while in the PG problem the number of selected neighbouring nodes is bounded, per each node. Furthermore, given two optimal solutions on two subgraphs, with common boundary vertices, of the planar graph, combining them together may not result in a feasible solution for PG on the whole graph. This is due to the possible infeasibility of local constraints of the boundary vertices of these two subgraphs. We overcome this major difficulty by introducing a new decomposition technique of planar graphs, which we call an $(\alpha ,\beta )$-decomposition, see Sect. 6.1 for details. This new technique is of independent interest and it may have further applications for the problems with constraints on vertices rather than on edges.

To obtain our PTAS for planar graphs on PG that may violate local quota constraints, we first use known rounding techniques (e.g., [13, 35]) to make all the coefficients polynomially bounded. Then, we design a dynamic programming approach to solve PG on any graph with a bounded tree-width tree decomposition. Finally, we combine a special tree decomposition of *k*-outerplanar graphs, called a nice tree decomposition, see [34], Baker’s shifting technique and our two-level dynamic programming approach for dealing with the global constraint, obtaining our PTAS.

Even when polluters’ cost functions are linear with a single parameter, simple monotonicity is not sufficient to turn our algorithms into truthful mechanisms (see e.g. Chapter 11 in [43]). This is because polluters’ utility functions have externalities—they are affected by their neighbours. Thus, we need to use general techniques to obtain truthful mechanisms: maximal in range mechanisms (for deterministic truthfulness) and maximal in distributional range mechanisms (for truthfulness in expectation). Our results are summarized in Table 1. As can be seen by this table our approximations are near best possible under appropriate complexity assumptions.

**Table 1:** Our results. TiE/DT: truthful in expectation/deterministic truthful mechanism. PG(poly) is PG with poly-size integer variables, PG(general) without this assumption

	General objective function	Linear objective function		
	Bounded Degree $\Delta$	Trees		Planar
Lower bound	$\varOmega \left( \frac{\Delta }{\log \Delta ^2}\right)$	NP-hard		Strongly NP-hard ($\delta$ violation)
PG(poly)	$O(\Delta )^\mathrm{a}$	FPTAS TiE	*O*(1) DT	PTAS ($\delta$ violation)
PG(general)	$O(\Delta )$ TiE $^\mathrm{b}$	FPTAS TiE [6]$^\mathrm{c}$		*O*(1) TiE [5]

$^\mathrm{a}$Monotone increasing obj. function.   $^\mathrm{b}$Piece-wise linear obj. function with one shift and an additional mild assumption.   $^\mathrm{c}$Running time is polynomial in *q*

## Literature Overview

An invaluable source of pollution control regulations comes from the managerial approaches in environment policies. The majority of literature in this field deals with symmetric information. This problem however shows a fundamental asymmetry between the regulatory bodies and pollutants. That is because the regulator (i.e. the government) and the rest of the players (i.e. the owners of pollution sources) have different incentives and therefore expressed through different utility functions, as explained in detail in Sect. 3. The research contributions considering environmental policy with asymmetric information and the diffusion nature of pollution have been limited until recently.

In order to control pollution, an incentive mechanism that is environmentally friendly and resource efficient needs to be designed and deployed by regulatory authorities. However, it is not obvious how to design such a mechanism in the presence of asymmetric information; just as Hurwicz [26] put it: the firms know that information will be used by the regulator to design a policy which will affect their profits. Hence, they have an incentive to manipulate reported information in order to influence the content of the policy. In this context, Farell [21] discusses the relevance of the Coase Theorem. This theorem basically asserts that bargaining will lead to an efficient outcome regardless of the initial allocation of property if negotiation and trade in presence of externality are possible and the transaction costs are sufficiently low. Considering the problems of incomplete information, that paper shows that voluntary negotiation does not lead to the first-best outcome that maximizes joint surplus in the presence of two-sided private information. That is to say, centralised economic institutions such as government control and intervention, and decentralised institutions such as bargaining and ownership rights, should be viewed as complementary to each other. Therefore, a necessary condition for the government when designing an optimal pollution control plan is the truthful information about firms.

Kwerel [38], Dasgupta et al. [15] and Spulber [50] have proposed mechanisms that implement truth telling by firms to maintain a mild level of pollution. Under this assumption the firms can communicate with the regulator but not with each other. In Kwerel’s scheme [38] firms are informed in advance that their messages will be translated into pollution taxes. The regulator issues a fixed number of transferable pollution licences and offers a subsidy for those licences which firms hold in excess of emission. Both the number of licences to be issued and the subsidy rate offered are calculated on the basis of the cost information provided by firms.

Kim and Chang [33] constructed an optimal incentive tax/subsidy scheme in an oligopoly market with pollution and suggested a differential damages mechanism, which leads to an optimal emission level. McKitrick [40] proposes that a Cournot Mechanism for pollution control under asymmetric information, in which a Nash Equilibrium exists, is stable and can be reached by iterative computations. Because firms may attempt to manipulate the pollution level allocation to their own advantage, the adjustment rule is exogenous and depends on the actions of the firms. The approach by Karp and Livernois [28] is related to that in Conrad and Wang [14]. The authors examined the steady-state properties of a tax adjustment mechanism in situations where the government has no information about firms’ abatement costs.

These prior studies provide an overall framework in the administrative approach to control pollution. However, those models are only a first level of approximation in characterizing the reality. Although, there is some literature studying an economics environment consisting of firms or countries with geographical distinction, few of them take the diffusion nature of air and water pollution into consideration. For instance, Petrosjan and Zaccour [44] study the problem of allocation over time of total cost incurred by countries in a cooperative game of pollution reduction. Segerson [48] develops a general incentive scheme for controlling nonpoint source pollution^1^ that considers the diffusion nature, in which rewards for environmental quality above a given standard are combined with penalties for substandard quality. Based on the work of Petrosjan and Zaccour [44], Belitskaya [11] develops an *n*-person network game model of emission reduction. Dorner et al. [19] create a multi-objective modeling system using Bayesian probability networks to study nonpoint source pollution. Both the work of Belitskaya [11] and Dorner et al. [19] are different from the setting of ours, in either model assumption or function settings. In addition to these works built on the network framework, Dong et al. [18] models the water pollution problem as a cost sharing problem on a tree network. However, none of the literature mentioned above takes into account the role of governments in pollution abatement, more specifically how to make policies assuming information asymmetry. A model that adequately takes both factors into account is what we need to tackle such problems in reality.

Few other papers have studied air pollution in relation to network models. Singh and Datta [49] use artificial neural network method to identify unknown pollution sources in the groundwater. Gianessi et al. [24] analyze the national water pollution control policies. And, finally, Trujillo and Hugh [54] study multi-objective air pollution monitoring network design. These papers use networks in a very different context from ours.

Turning into current practice, emission trading is a market-based approach used to control pollution by providing economic incentives for achieving reductions in the emissions of pollutants. Various countries have adopted emission trading systems as one of the strategies for mitigating climate-change by addressing international greenhouse-gas emission [52]. Usually a governmental body sets a limit or cap on the amount of a pollutant that may be emitted. The limit or cap is allocated and/or sold by the central authority to firms in the form of emission permits which represent the right to emit or discharge a specific volume of the specified pollutant [51]. Permits (and possibly also derivatives of permits) can then be traded on secondary markets. For example, the European Union Emissions Trading Scheme (EU ETS) trades primarily in European Union Allowances (EUAs), the Californian scheme in California Carbon Allowances, the New Zealand scheme in New Zealand Units and the Australian scheme in Australian Units [53]. Firms are required to hold a number of permits (or allowances or carbon credits) equivalent to their emissions. The total number of permits cannot exceed the cap, limiting total emissions to that level. Firms that need to increase their volume of emissions must buy permits from those who require fewer permits [51, 52]. Currently a simple auction mechanism for selling EUAs is adopted in Europe, see, e.g., [3]. Furthermore in order to limit the automobile pollution, governments use policies of car taxation [22, 27]. A radical transport policy introduced in the UK and first applied in Central London resulting in 19% reduction of CO$_2$ emissions (see Table 2 in [9]).

## Model and Applications

We first describe the general model of a Pollution Game (PG) and then explain how our two suggested applications fit into it. We are given an area of pollution sources (e.g., factories, cars) each owned by an agent. The government acts as a regulator restricting the levels of emitted pollution, while aiming to maximize the social welfare.

More formally, we are given a weighted digraph $G=(V,E)$, where *V* is the set of *n* pollution sources, also called players or agents, and *E* represents the set of neighbouring nodes, i.e., $(u,v) \in E$ if and only if an amount of pollution can be transferred by *u* to *v*. In this model no geometric assumptions are made. For each $(u,v) \in E$, the weight $w_{(u,v)} = w_{uv}$ denotes the pollution transfer factor from node *u* to *v*. Without loss of generality we may suppose that $w_{uv} \in (0,1]$, $\forall (u,v)\in E$. Intuitively, $w_{uv}$ is percentage of the pollution emitted at *u* that reaches *v*.

The government needs to decide the number of licences $x_v \in \{0,\ldots ,q_v\}$ that can be sold to each source $v \in V$, where $q_v \in {\mathbb {N}}$ is the total number of licences that can be issued per region of the given area. Furthermore, the government sets a bound on the total pollution quota discharged to the environment to be equal to *p*, which corresponds to the total number of licences. This is called the *global constraint*:

1 $$\begin{aligned} \sum _{v\in V} x_v \le p \end{aligned}$$

Each agent *v* has a non-decreasing benefit function $b_v : \{0,\ldots ,q_u\} \longrightarrow \mathbb {R}_{\ge 0}$, where $b_v(x_v)$ is a concave increasing function (economic diminishing marginal utility phenomenon)^2^ with $b_v(0)=0$, representing the benefit incurred by *v*. Each agent *v* also has a non-decreasing damage function $d_v: \mathbb {R}_{\ge 0} \longrightarrow \mathbb {R}_{\ge 0}$, representing that the damaging effect of more emitted pollution is accelerating. Player *v*’s total welfare $r_v$ is *v*’s benefit minus his damage cost:

2 $$\begin{aligned} r_v=b_v(x_v)-d_v\left( x_v+\sum _{u\in \delta ^{-}_G(v)} w_{uv}x_u\right) \end{aligned}$$

where $\delta ^{-}_G(v) = \{u \in V: (u,v) \in E\}$, $\delta ^{+}_G(v) = \{u \in V: (v,u) \in E\}$. We assume that the government decides on the allowable local level of pollution $p_v$, for every $v \in V$. This imposes the following *local constraints* called the local level of pollution of $v \in V$:

3 $$\begin{aligned} x_v+\sum _{u\in \delta ^{-}_G(v)} w_{u v}x_u\le & {} p_v \end{aligned}$$

4 $$\begin{aligned} x_v\le & {} q_v \end{aligned}$$

The optimization problem of social welfare maximization can be formulated in the general form by the following integer program:

5 $$\begin{aligned} \max&R(x)=\sum _{v\in V} \Bigg ( b_v(x_v)-d_v\Bigg ( x_v+\sum _{u\in \delta ^{-}_G(v)} w_{u v}x_u \Bigg ) \Bigg ) \end{aligned}$$

6 $$\begin{aligned} \text{ s.t. }&\sum _{v \in V} x_v \le p \end{aligned}$$

7 $$\begin{aligned}&x_v+\sum _{u\in \delta ^{-}_G(v)} w_{uv}x_u \le p_v, \,\,\, \forall v \in V \end{aligned}$$

8 $$\begin{aligned}&x_v \in \{0,1, \ldots , q_v\}, \,\,\, \forall v \in V \end{aligned}$$

We call this problem PG with integer variables, if $x_v \in \mathbb {Z}$, or with binary variables, if $x_v\in \{0,1\}$. For an instance *I* of PG, |*I*| denotes the number of bits to encode *I*, and if $q\in poly(|I|)$, where $q=\max _{v\in V}\{q_v\}$+1, we call this problem PG with polynomial size integer variables.

We note here, that the game theoretic ingredients of our PG model will be defined in Sect. 3.3.

### Application 1: Regulation of Pollution Sources

In our first application the pollution sources are factories and the agents in this case are their owners. In the weighted digraph $$G=(V,E)$$, *V* is the set of *n* factories and *E* is the set of neighbouring ones, i.e., $$(u,v) \in E$$ if and only if the pollution emitted by *u* affects *v*. The weight $$w_{(u,v)} = w_{uv}$$ denotes a discount factor of the pollution discharged by agent *u* affecting its neighbour *v*. This can be intuitively understood as a percentage of pollution emitted at *u* that reaches, via air, *v*. The government has to decide which factories must remain open and which must be shut down in order for the local and global constraints to be fulfilled. This fact is denoted by the value of $$x_v$$. If the owner of factory $$v\in V$$ is given a licence then we set $$x_v=1$$, otherwise the factory must be shut down and we set $$x_v=0$$. As a result, in this first application we assume that $$x_v \in \{0,1\}$$ and $$q_v=1,$$$$\forall v \in V$$, and $$b_v : \{0,1\} \longrightarrow \mathbb {R}_{\ge 0}$$. In this case the global constraint corresponds to the maximum number of awarded licences, or, equivalently, the maximum number of factories that can remain open in the whole area.

Although the pollution sources might not emit the same amount of effluents, they are treated as equal. That is because the imposed constraints by the government in every subarea take into account the total amount of effluents emitted by the pollution sources and not by each one independently, i.e., the decision of shutting down a factory depends also on the structure of the neighborhood graph.

### Application 2: Allocation of Pollution Licences

In the second application formulated by the above convex program we consider an area of *n* cities each one administered by its mayor, who is the agent in this case. In every city statistical observations are used to measure the car traffic to the neighboring cities. More precisely we consider a network represented by a weighted digraph $G=(V,E,w)$, where *V* is the set of *n* agents (mayors of the cities), *E* is the set of roads connecting cities such that $(u,v) \in E$ if and only if *u* and *v* are neighboring cities. Then, $w: E \rightarrow {\mathbb {R}}$ represents the percentage of cars entering a city from a neighboring one, i.e., $w_{uv}$ denotes the percentage of cars driving from *u* to *v* in some time interval measured by observations. The duty of the regulator is to allocate a number of licences to the agents (mayors) such that the total welfare is maximized while fulfilling a number of constraints. The agent with $x_u$ licences gains a benefit of $b_u(x_u)$ which is a monetary income coming from selling these $x_u$ licences to car drivers, one licence per car. Our model does not model this explicitly but just assumes for simplicity that all $x_u$ licences are sold.

Naturally a percentage of cars with licences from city *u* remains in *u* and the rest is split and drives into the neighboring cities. We denote by $w_u$ the percentage of cars remaining in *u* and $w'_{vu}$ the percentage of cars entering *u* from neighbouring city *v*. The maximum number of cars (maximum number of licences) allowed at any moment in city *u* is bounded by $p'_u$ also given in the input. This is represented by the local constraint: $w_u x_u+\sum _{v \in \delta (u)}w'_{vu}x_v \le p'_u$. If $w_u \ne 0$ the last inequality can equivalently be written as $x_u+\sum _{v \in \delta (u)}w_{vu} x_v \le p_u$, where $w_{vu}=w'_{vu}/w_u$ and $p_u=p'_u/w_u$.

Planar graphs are close to real applications, and it is natural to study our second application on planar networks [56]. Imagine a collection of cities (each being a contiguous geographic area) and roads connecting them. This defines a planar map where we only consider edges (roads) between neighbouring cities, which implies a planar graph. We disregard other roads and we consider only frequent driving patterns in a time interval measured by observations. They correspond to frequent commuters, e.g., between house and work, which typically are neighbouring cities.

In the following sections we assume that $b_v$ and $d_v$ are both linear functions with slopes $s^0_v$ and $s^1_v$ respectively, i.e. $b_v(x)=s^0_vx$ and $d_v(y)=s^1_vy$, for any $v\in V$. Let $\omega _v=s^0_v-s^1_v-\sum _{u\in \delta ^{+}_G(v)}s^1_uw_{vu}$. The social welfare function is:

9 $$\begin{aligned} \begin{aligned} R(x)&=\sum _{v\in V}b_v(x_v)-d_v\left( x_v+\sum _{u\in \delta ^{-}_G(v)}w_{uv}x_u\right) \\&=\sum _{v\in V}s^0_vx_v-s^1_v\left( x_v+\sum _{u\in \delta ^{-}_G(v)}w_{uv}x_u\right) \\&=\sum _{v\in V}\omega _vx_v. \end{aligned} \end{aligned}$$

### Basic Definitions

Let $I=(G,\mathbf {b,d,p,q})$ be an instance of PG, where ${\mathbf {b}}=(b_v)_{v \in V}$, ${\mathbf {d}}=(d_v)_{v \in V}$, ${\mathbf {p}}=(p_v)_{v \in V}$ and ${\mathbf {q}}=(q_v)_{v \in V}$ ($b_v$ is assumed private information of *v* and the other parameters public). Let ${\mathcal {I}}$ be the set of all instances, and ${\mathcal {X}}$ the set of feasible allocations. Given a digraph $G=(V,E)$ the undirected graph $G^{un}=(V,E^{un})$ is such that $E^{un} = \{(u,v) : (u,v) \in E \text{ or } (v,u) \in E\}$.

A mechanism $$\phi =(X,P)$$ consists of an allocation $$X:{\mathcal {I}}\rightarrow {\mathcal {X}}$$ and payment function $$P:{\mathcal {I}}\rightarrow \mathbb {R}^{|V|}_{\ge 0}$$ (*X*(*I*) satisfies (6)–(8)). For any vector *x*, $$x_{-u}$$ denotes vector *x* without its *u*th component. We also denote by $$(y,x_{-u})$$ vector *x* that has *y* at position *u*, in particular, $$(x_u, x_{-u})=x$$. Note, $$r_v(X(I))=b_v(X_v(I))-d_v(X_v(I)+\sum _{u\in \delta ^{-}_G(v)}w_{uv}X_u(I))$$ is the welfare of player *v* under *X*(*I*). A mechanism $$\phi =(X,P)$$ is truthful, if for any $$b_{-v}$$, $$b_v$$ and $$b'_v$$, $$r_v(X(b_v,b_{-v}))-P_v(b_v ,b_{-v})\ge r_v(X(b'_v,b_{-v}))-P_v(b'_v,b_{-v})$$. A randomized mechanism is truthful in expectation if for any $$b_{-v}$$, $$b_v$$ and $$b'_v$$, $$\mathbb {E}(r_v(X(b_v,b_{-v}))-P_v(b_v ,b_{-v}))$$$$\ge \mathbb {E}(r_v(X(b'_v,b_{-v}))-P_v(b'_v,b_{-v}))$$, where $$\mathbb {E}(\cdot )$$ is over the algorithm’s random bits. Note, that the utility of player *v* is defined as $$u_v=r_v(X(b_v,b_{-v}))-P_v(b_v ,b_{-v})$$ and the expected utility as $$\mathbb {E}(r_v(X(b_v,b_{-v}))-P_v(b_v ,b_{-v}))$$.

A mechanism is individually rational if each agent *v* has non-negative utility when he declares $b_v$, regardless of the other agents’ declarations.

In the following we will denote by $OPT^{fr}_G(PG)$ the value of the optimal fractional solution of PG on *G*. Similarly $OPT^{in}_G(PG)$ denotes the optimal integral solution. The integrality gap of PG on *G* is defined as $\frac{OPT^{fr}_G(PG)}{OPT^{in}_G(PG)}$. The approximation ratio of an algorithm ${\mathcal {A}}$ with respect to $OPT^{in}_G(PG)$ ($OPT^{fr}_G(PG)$ respectively) is $\rho ^{in}({\mathcal {A}})=\frac{OPT^{in}_G(PG)}{R({\mathcal {A}})}$ ($\rho ^{fr}({\mathcal {A}})=\frac{OPT^{fr}_G(PG)}{R({\mathcal {A}})}$), where $R({\mathcal {A}})$ is the objective value of the ${\mathcal {A}}$’s solution. Unless stated otherwise, the approximation $\rho$ will be with respect to $OPT^{fr}_G(PG)$. An FPTAS (PTAS, EPTAS, respectively)^3^ for a problem ${\mathcal {P}}$ is an algorithm ${\mathcal {A}}$ that for any $\epsilon >0$ and any instance *I* of ${\mathcal {P}}$, outputs a solution with the objective value at least $(1-\epsilon ) OPT^{in}_I({\mathcal {P}})$ and terminates in time $poly(\frac{1}{\epsilon },|I|)$ ($(\frac{1}{\epsilon }|I|)^{g(\frac{1}{\epsilon })}$ and $g(\frac{1}{\epsilon })poly(|I|)$, respectively), where *g* is a function independent from *I*. We also let $[n]=\{1,\ldots ,n\}$.

*Algorithms for packing problems* A linear constraint $a x \le b$ with $x \in \mathbb {N}_{\ge 0}^{n}$ an integer vector, and $a, b \in \mathbb {R}_{\ge 0}^{n}$ are vectors, is called a packing constraint. A linear (respectively convex submodular) maximization programming problem with packing constraints is called a linear (respectively convex) packing programming problem. Linear refers to a linear objective function and convex submodular to a convex submodular objective function. A *k*-column-sparse packing integer program is one in which each variable *j* participates in at most *k* constraints, where

$$\begin{aligned} \gamma _k=\min \left\{ 2k^2+2, 8k,\frac{k}{\left( 1-\frac{1}{k}\left( 1+\left( \frac{2}{k}\right) ^{\frac{1}{3}}\right) \right) ^k}\right\} = (e+o(1))k=O(k). \end{aligned}$$

For a given *k* we have the following propositions where the first holds for linear and the second for convex functions:

#### Proposition 1

[10, 45] There is a polynomial time deterministic algorithm for *k*-column-sparse linear packing programming problem with binary variables, achieving the approximation ratio $\rho ^{fr}=\gamma _{k}$.

#### Proposition 2

[10] There is a polynomial deterministic algorithm for *k*-column-sparse convex submodular packing programming problem with binary variables, achieving the approximation ratio $\rho ^{fr}=\frac{e\gamma _{k}}{e-1}$.

#### Proposition 3

[39] For any linear packing programming problem, if there is a polynomial deterministic algorithm with the approximation ratio $\rho ^{fr}$ for this problem, then there is a polynomial, randomized, individually rational, $\rho ^{fr}$-approximation mechanism for the same problem that is truthful in expectation.

## Hardness

Observe that PG is weakly NP-hard even on stars and even without the global constraint with linear valuation functions. Consider the star where the central node *u* is connected to nodes $v_1, v_2, \ldots , v_n$ with valuations $\omega _{v_1}, \omega _{v_2}, \ldots , \omega _{v_n}$ and the edges that connect them have weights $w_{uv_1}, w_{uv_2}, \ldots , w_{uv_n}$ respectively. Then PG on this instance (omitting the local constraints of all the nodes except *u*) can be formulated by the following ILP:

$$\begin{aligned} \max&\omega _u x_u + \sum _{i=1}^n \omega _{v_i} x_{v_i}\\ \text{ s.t. }&x_u + \sum _{v_i\in \delta ^{-}_G(u)} w_{v_iu}x_{v_i} \le p_u\\&x_{v_i} \in \{0,1\}, \,\,\, \forall v_i \in \delta ^{-}_G(u) \end{aligned}$$

**Fig. 1:**
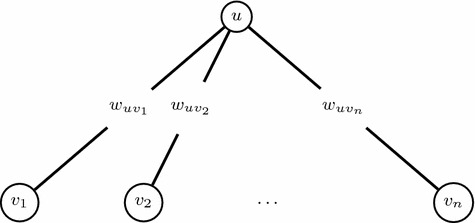
An instance of PG on a star

The above ILP is the Knapsack problem [55]. In the Knapsack problem we are given *n* items each having a weight $w_i$ and a value $v_i, \forall i=1, \ldots , n$. We are also given capacity *W* and we are asked to choose those items whose total value is maximized under the constraint that the capacity is not exceeded. In the instance described above the weights on the edges are the weights of the items and the values of the nodes are the values of the items. Knapsack in known to be weakly NP-hard, thus PG on stars is also weakly NP-hard (Fig. 1).

We also note that inequality (6) can also be written as equality

10 $$\begin{aligned} \sum _{u \in V} x_u = p \end{aligned}$$

since the upper limit of the total amount of the pollution is controlled by the government. If $\sum _{v \in V} x_v < p$ in the final allocation, then the government can simply set *p* equal to $\sum _{v \in V} x_v$ without any changes. However, for computational issues, these two representations lead to different computational complexity of the problem. If inequality (6) is replaced by (10), then even finding a feasible solution of PG is NP-complete. Therefore, unless stated otherwise, we always suppose inequality (6) as a constraint of PG.

### Theorem 1

Finding a feasible solution which satisfies constraints (10) and (7) to PG when $p_v=1 \forall v \in V$ and $w_{uv}>0$ for any $(u, v)\in E$ is NP-complete.

### Proof

It is straightforward that the problem is in NP. Consider now a formula of monotone 1-in-3 SAT where an instance of this problem consists of *n* Boolean variables and *m* clauses. A YES instance is one in which an assignment to its Boolean variables is such that exactly one literal from each clause is true. The problem is known to be NP-Complete even when there are no negations [47]. The following proof is inspired by the reduction of 3-SAT to Independent Set (p. 248 [16]).

**Fig. 2:**
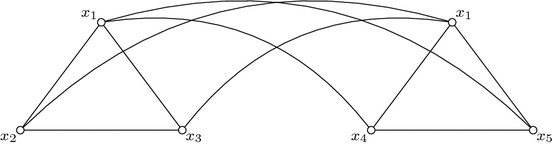
Illustration of the reduction

Let us represent a clause, say $(x \vee y \vee z)$, by a triangle with vertices labeled *x*, *y*, *z*. Repeat this construction for all clauses. Next consider one of the literals, say *x*, which appears in *k* clauses $C_{i_1}, \ldots , C_{i_k}$. Let $Tr_{i_1}, \ldots , Tr_{i_k}$ be the triangles of the clauses $C_{i_1}, \ldots , C_{i_k}$ respectively. Then connect *x* of $Tr_{i_1}$ with all the vertices of $Tr_{i_2}, \ldots , Tr_{i_k}$ except those labeled with *x*. Repeat this construction for *x* in all these triangles and for all literals. For example consider the formula $\varPhi =(x_1 \vee x_2 \vee x_3)\wedge (x_1 \vee x_4 \vee x_5)$ (see Fig. 2). First construct the triangles labeled $x_1,x_2,x_3$ and $x_1,x_4,x_5$ for the two clauses respectively. Then connect vertex $x_1$ of the first clause with the vertices $x_4$ and $x_5$ of the second clause. In the same way connect vertex $x_1$ of the second clause with the vertices $x_2$ and $x_3$ of the first clause.

Consider now an instance of 1-in-3 SAT which is satisfiable and let $G=(V,E)$ be the corresponding graph constructed as above, letting $p_u=1$ and $p=m$ for every vertex $u \in V$. Suppose that we have a truth assignment which satisfies all the clauses. Then this means we choose *p* vertices in *G* without violating any of the constraints. Indeed if two vertices have the same label then they are not connected. If they have different labels, say *x* from clause $C_1$ and *y* from clause $C_2$ and they are connected, this is because their corresponding clauses have a common literal, either *x* or *y*. Thus if one of them has value true, the other will have value false for the formula to be satisfiable. Finally if two vertices belong to the same clause, only one of them will have the value true.

Suppose now that there is a solution for graph *G* with *m* vertices when $$p_v=1, $$$$\forall v \in V$$ (recall that $$p = m$$). Then setting the literal in the set $$\{v:x_v=1\}$$ is a solution of 1-in-3 SAT. The argument is as follows, in each triangle, there is exactly one vertex such that its value is one since at most one vertex in each triangle can be selected and there are *m* triangles and $$p=m$$. By the construction of *G*, these vertices cannot be connected to each other (and thus, form a solution of 1-in-3 SAT), due to (7) and the fact that $$p_v=1, \forall v \in V$$. $$\square $$

### Theorem 2

It is strongly NP-hard to find an optimal solution to Pollution Game (PG) when $p_v$ is any constant number $\ge 1$ , $b_v(x_v)$ is linear and $d_v(y)$ is piecewise linear (with two pieces) $\forall v \in V$ and $w_{vu}$ is positive constant for any $(v, u)\in E$.

### Proof

First we only consider undirected graphs, however, our reduction also applies to directed graphs. Let $G=(V,E)$ be a graph with degree $d(G) \le d$. Next construct a bipartite graph $G'=(V',U',E')$ with $|V'|=|V|$ and $|U'|=|E|$, where each vertex of $V'$ corresponds to a vertex of *V* and each vertex of $U'$ corresponds to an edge of *E*. Connect a vertex $v \in V'$ with a vertex $u\in U'$ if the corresponding vertex of *v* is incident to the corresponding edge of *u* in G. It can easily be seen that every $v \in V'$ has degree at most *d* and every $u \in U'$ has degree 2. Let $b_v(x_v)=x_v$ and $d_v(x_v)=0, \forall v \in V'$. Furthermore, for any $u\in U'$, let $b_u(x_u)=0$ and $d_u(y)=\frac{|V|(y-\max \{w_{vu},w_{v'u}\})}{\min \{1,w_{vu}+w_{v'u}\}-\max \{w_{vu},w_{v'u}\}}$ if $y > \max \{w_{vu}, w_{v' u}\}$ and $d_u(y)=0$ otherwise, where $(v,u)\in E'$ and $(v',u)\in E'$. The intuition behind the definition of this damage function is that it basically allows the second claim below to hold.

Let *W* be an independent set of *G*. Let $|W|=k \le p$. Then the welfare of *W* for PG on $G'$ is *k*. Suppose now there is a better solution $W'$ with larger welfare for PG. We declare the following two claims:

### Claim

$$W' \cap U'= \emptyset $$

If $W' \cap U'\ne \emptyset$, suppose a vertex $u\in U'$ is included in $W'$, then $r_u\le 0$. Hence, removing *u* from $W' \cap U'$ will not decrease the total welfare.

### Claim

Any two vertices $u,v \in W'$ are not connected to the same vertex in $U'$.

If there exist two vertices $u, v \in W'$ that are connected to the same vertex in $U'$, suppose they are connected to $u'\in U'$. Then we know $r_u=r_v=1$. However, since for the local level of pollution in $u'$ is $y\ge w_{vu}+ w_{v' u}>\max \{w_{vu}, w_{v' u}\}$, we have $r_{u'}= -d_{u'}(y)\le -|V|$. Hence, the total welfare achieved by $W'$ is at most $|W'\cap V'|-|V|\le 0< k$ . Removing either *u* or *v* from $W'$ will increase welfare by $|V|-1$.

Therefore, $W'$ corresponds to an independent set in *G* with size larger than |*W*|. Thus, any independent set *W* gives a welfare of |*W*| in $G'$. As a consequence, if we can find a solution of PG in $G'$ with welfare at least *k*, then we can easily find an independent set in *G* of size at least *k*. And, in the other direction, an independent set in *G* of size *k* corresponds directly to a PG solution in $G'$ with welfare *k*. $\square$

From [23] it is strongly NP-hard to find the maximum independent set on a planar graph with degree at most 3.

### Corollary 1

For a planar graph $G=(V,E)$ with degree at most 3, the problem of finding an optimal solution in PG setting as in Theorem 2 is strongly NP-hard.

### Proof

For any planar graph $G=(V,E)$, the constructed graph $G'=(V',U',E')$ in the proof of Theorem 2 is planar. To see this, just add one vertex to the center of each edge in *G* representing the edge vertex in $U'$. The resulting graph is planar and the same as $G'$. The corollary follows from the reduction in the proof of Theorem 2. $\square$

In the next theorem we use a by now commonly used complexity theoretic Unique Games conjecture, see [31].

### Theorem 3

PG is Unique Games-hard to approximate within $n^{1-\epsilon }$ and within $\frac{\Delta }{\log ^2\Delta }$ for graph *G* with degree $\Delta$ when $p_v$ is any constant number $\ge 1$ , $b_v(x_v)$ is linear and $d_v(y)$ is piecewise linear (with two pieces) $\forall v \in V$ and $w_{vu}$ is positive constant for any $(v, u)\in E$.

### Proof

According to [32], maximum independent set is Unique Games-hard to approximate within $n^{1-\epsilon }$ in general graphs and within $\frac{\Delta }{\log ^2\Delta }$ for graph *G* with degree $\Delta$. The theorem follows from the reduction in the proof of Theorem 2. $\square$

### Theorem 4

There is no EPTAS for PG with binary variables on the directed planar graph $G=(V,E)$ when $b_v$ and $d_v$ are both linear functions, for any $v\in V$.

### Proof

**Fig. 3:**
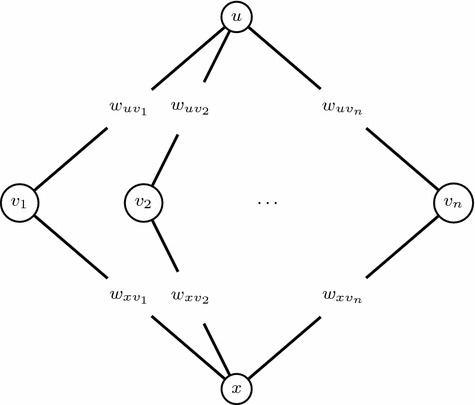
The two dimensional knapsack on an instance of a planar graph

Consider PG on the following simple planar graph. There are $n+2$ vertices labeled as $\{o_1,o_2,1,2,...,n\}$ and the edge set $E=\{(i, o_j),i\in [n],j\in \{1,2\}\}$ with weights $w_{io_j}$, $i\in [n],j\in \{1,2\}$. For any two dimensional knapsack problem, there exists an instance of PG with binary variables without the global constraint on such a simple graph exactly corresponding to this two dimensional knapsack problem. According to [37], there is no EPTAS for two dimensional knapsack. Hence, there is no EPTAS for PG on this simple planar graph (Fig. 3). $\square$

## Directed Trees

We present a truthful in expectation FPTAS for PG on directed trees by a two level dynamic programming approach and a 3-approximation deterministic truthful mechanism.

### Truthful in Expectation Mechanisms

A digraph *G* is called a *directed tree* if the undirected graph $G^{un}$ is a tree. We consider rooted trees where arcs are directed towards the leaves. We obtain our truthful in expectation FPTAS for PG with binary variables on any directed trees by a two-level dynamic programming (DP) approach. The first bottom–up level is based on a careful application of the standard single-dimensional knapsack FPTAS. The second level is by an interesting generalization of an FPTAS of [12] for a special multiple choice multi-dimensional knapsack problem with a constant number of constraints most of which have *poly*(|*I*|) size of coefficients. This FPTAS generalizes the results in [12], where the authors consider the one dimensional knapsack problem with cardinality constraint.

Consider the following instance *I* of a Special multiple choice and multi dimensional Knapsack Problem (denoted as SKP):

$$\begin{aligned} \max&C(x)=\sum _{j\in [J]}\sum _{k\in [K]}C_{jk}x_{jk} \qquad \qquad \qquad (SKP)\\ \text{ s.t. }&\sum _{j\in [J]}\sum _{k\in [K]}A_{ijk}x_{jk}\le B_i,\,\,\, \forall i\in [N]\\&\sum _{k\in [K]}x_{jk}\le 1 \,\,\, \forall j \in [J] \\&\sum _{j\in [J]}\sum _{k\in [K]}A'_{jk}x_{jk}\le B'\\&x_{jk}\in \{0,1\}, \,\,\, \forall j\in [J],\, k\in [K] \end{aligned}$$

where:

- *J* is the number of items available for selection,
- *K* denotes the number of different classes of items where at most one item can be chosen from each class,
- $N=O(1)$ is the number of dimensions of the constraints or items,
- $C_{jk}$ denotes the profit of item *j* from class *k*,
- $$B_i=poly(|I|)$$, $$\forall $$$$i\in [N]$$ is the capacity (size) of the *i*th dimension,
- $A_{ijk}$ is the size of *j*th item in dimension *i* from class *k* and
- $A'_{jk}$ is the size of the *j*th item from class *k* of dimension $N+1$.

Without loss of generality suppose all the parameters in the above knapsack problem are integers and $$A_{ijk}\le B_i=poly(|I|)$$, $$\forall $$$$i\in [N]$$, $$j\in [J]$$, $$k\in [K]$$. Let $$C=OPT(I)$$ and $$B=\max _{i\in [N]}{B_i}$$.

#### Lemma 1

There is a pseudo polynomial optimal algorithm for SKP, terminating in $O(CJKB^{N})$ time.

#### Proof

We use a similar technique used to derive an FPTAS for the Knapsack problem (Chapter 8 in [55]). In order to solve the above LP we convert it into a minimization LP. To do so we take the last constraint of the original LP and make it the objective of the new one and we also take the original objective, we make it as constraint of the new LP setting it equal to *M*. Each of of the other constraints $i \in [N]$ (i.e. every other dimension) is set to be equal to $\ell _i$. Let now $\ell =(\ell _1,\ell _2,\ldots ,\ell _N)$. We now have the following linear program:

$$\begin{aligned} \min&h_s(M,\ell )=\sum _{j\in [s]}\sum _{k\in [K]}A'_{jk}x_{jk} \\ \text{ s.t. }&\sum _{j\in [s]}\sum _{k\in [K]}C_{jk}x_{jk}=M\\&\sum _{j\in [s]}\sum _{k\in [K]}A_{ijk}x_{jk}=\ell _i \,\,\, \forall i \in [N]\\&\sum _{k\in [K]}x_{jk}\le 1, \forall j \in [s]\\ \end{aligned}$$

Initially, $h_0(M,\ell )=+\infty$, for all *M*, $\ell$. Then set $h_0(0,0)=0$. As a result, the recursion can be calculated as follows:

$$\begin{aligned} h_s(M,\ell )=\min \{h_{s-1}(M,\ell ),\min _{k\in [K]}\{ h_{s-1}(M-C_{sk}, (\ell _i-A_{isk})_{i\in [N]})\}\}. \end{aligned}$$

Then the optimal solution of SKP is

$$\begin{aligned} \max _{M\le C,\ell _i\le B_i, i\in [N]}\{M: h_{J}(M,\ell )\le B'\} \end{aligned}$$

Note that the running time of this dynamic programming approach is $O(CJKB^{N})$. $\square$

Let $C(z)=\max _{j\in [J],k\in [K]}C_{jk}$. Note that

$$\begin{aligned} \frac{OPT^{in}(I)}{J}\le C(z)\le OPT^{in}(I) \end{aligned}$$

We now scale all the coefficients in the objective function *C*(*x*). Let

$$\begin{aligned} {\tilde{C}}_{jk}=\left\lceil \frac{C_{jk}J}{C(z)\epsilon }\right\rceil \le \frac{C_{jk}J}{C(z)\epsilon }+1, \forall j\in [J], k\in [K] \end{aligned}$$

The optimal value ${\tilde{C}}$ of scaled SKP is then upper bounded by

$$\begin{aligned} \frac{CJ}{C(z)\epsilon }+J\le \frac{J^2}{\epsilon }+J \end{aligned}$$

Consider the dynamic programming approach running on the scaled SKP as ${\mathcal {A}}^{scaled}$. Then we have

#### Theorem 5

${\mathcal {A}}^{scaled}$ is an FPTAS for SKP, terminating in $O(\frac{J^3KB^N}{\epsilon })$ time.

#### Proof

We only need to show the approximation part i.e. that the optimal solution returned is within $1-\epsilon$ of the optimal value of SKP, since the running time straightforwardly follows from the running time of the dynamic programming approach

$$\begin{aligned} O\left( {\tilde{C}}JKB^N\right) =O\left( \left( \frac{J}{\epsilon }+1\right) J^2KB^N\right) =O\left( \frac{J^3KB^N}{\epsilon }\right) \end{aligned}$$

Let ${\tilde{S}}$ and *S* denote the optimal solution of the scaled and the original SKP respectively. Note that ${\tilde{S}}$ is a feasible solution to the original SKP. By scaling,

11 $$\begin{aligned} \frac{({\tilde{C}}_{jk}-1)C(z)\epsilon }{J}\le C_{jk}\le \frac{{\tilde{C}}_{jk}C(z)\epsilon }{J} \end{aligned}$$

Then

$$\begin{aligned} \begin{aligned} C(S)-C({\tilde{S}})&\le \frac{C(z)\epsilon }{J} ({\tilde{C}}(S)-{\tilde{C}}({\tilde{S}})+|S|)\\&\le \frac{C(z)\epsilon |S|}{J} \le C(z) \epsilon \le C(S)\epsilon \end{aligned} \end{aligned}$$

where the last inequality comes from $|S|\le J$. $\square$

We will also need the following tool from mechanism design for packing problems. An integer linear packing problem with binary variables is a problem of maximising a linear objective function over a set of linear packing constraints, i.e., constraints of form $a \cdot x\le b$ where $x \in \{0,1\}^{n}$ is a vector of binary variables, and $a, b \in \mathbb {R}_{\ge 0}^{n}$.

#### Proposition 4

[20] Given an FPTAS for an integer linear packing problem with binary variables, there is a truthful in expectation mechanism that is an FPTAS.

We first present an FPTAS on directed trees without global constraint which captures our main technical ingredients.

#### FPTAS Without Global Constraint

The algorithm uses a dynamic programming approach and the FPTAS for knapsack problem as a subroutine. Note that on a star, any instance of knapsack can be reduced to a PG instance without global constraints. Thus, an FPTAS is the best we can hope for such PG unless $P=NP$.

We keep four values for each $v\in V$. Suppose that the father of *v* is $v'$. Let $M^{v'\text {in}}_{v\text {in}}$ denote the optimal value of PG on the subtree rooted at *v* when both $v'$ and *v* are selected in the solution. Similarly, we also have $M^{v'\text {in}}_{v\text {out}}$, $M^{v'\text {out}}_{v\text {in}}$ and $M^{v'\text {out}}_{v\text {out}}$. Let $u_i$, $i=1,2,...,n_v$ denote the children of *v*. Suppose $M^{v\text {in}}_{u_i\text {in}}$, $M^{v\text {in}}_{u_i\text {out}}$, $M^{v\text {out}}_{u_i\text {in}}$ and $M^{v\text {out}}_{u_i\text {out}}$ have been calculated, for any $i=1,...,n_v$. Some of them may be undefined due to infeasibility. We will calculate now $M^{v'\text {in}}_{v\text {in}}$. Recall that $\omega _v=s^0_v-s^1_v-\sum _{u\in \delta ^{+}_G(v)}s^1_uw_{vu}$. Observe that $M^{v'\text {in}}_{v\text {in}}$ is equal to the optimal value of the following knapsack $(IP_1)$, where $M^{v\text {in}}_{u_i\text {in}}$ and $M^{v\text {in}}_{u_i\text {out}}$ have finite values (otherwise we remove them):If this knapsack problem has a feasible solution, we get the value $M^{v'\text {in}}_{v\text {in}}$, otherwise we set $M^{v'\text {in}}_{v\text {in}}$ to be undefined. Similarly we calculate $M^{v'\text {in}}_{v\text {out}}$, $M^{v'\text {out}}_{v\text {in}}$ and $M^{v'\text {out}}_{v\text {out}}$. Thus, if we can calculate an optimal solution at each step, this solution will be obtained by the above DP approach. For knapsack with $n_v$ variables, there is an FPTAS. Hence, at each step we get an approximate value ${\bar{M}}^{v'\text {in}}_{v\text {in}}\ge (1-\epsilon )M^{v'\text {in}}_{v\text {in}}$ in time polynomial in $n_v$ and $\frac{1}{\epsilon }$ by knapsack’s FPTAS. In a similar way we compute approximately the other three values. Thus, in the final solution, ${\bar{M}}_{root}\ge (1-\epsilon )^k M_{root}$, where *k* is the number of levels of the tree and $M_{root}$ is the optimal value of PG without global constraints, terminating in $poly(|I|, \frac{1}{\epsilon })$ time where |*I*| is the input size. If we let $1-\epsilon '=(1-\epsilon )^k$, we have that $\epsilon =\varTheta (\frac{\epsilon '}{k})$. The running time is $poly(|I|,\frac{k}{\epsilon '})=poly(|I|,\frac{1}{\epsilon '})$ due to $k\le |I|$, giving an FPTAS for PG without global constraint. $\square$

#### FPTAS with Global Constraint

Suppose without loss of generality that $p \le n$, otherwise let $p=n$. For each vertex *v*, we will keep 4*p* values. Suppose that the father of *v* is $v'$. Let $M^{v'\text {in}}_{v\text {in}}(s)$ denote the optimal value of PG on the subtree rooted at *v* when both $v'$ and *v* are selected in the solution, and the total pollution level allocated to the subtree rooted at *v* is no more than *s*, $s=0,1,\ldots ,p$. Similarly, we also have $M^{v'\text {in}}_{v\text {out}}(s)$, $M^{v'\text {out}}_{v\text {in}}(s)$ and $M^{v'\text {out}}_{v\text {out}}(s)$. Let $u_i$, $i\in [n_v]$ denote the children of *v*. Suppose that $M^{v\text {in}}_{u_i\text {in}}(s)$, $M^{v\text {in}}_{u_i\text {out}}(s)$, $M^{v\text {out}}_{u_i\text {in}}(s)$ and $M^{v\text {out}}_{u_i\text {out}}(s)$ have been calculated, for any $i \in [n_v]$ and $s=0,1,\ldots ,p$. Some of them may be undefined due to infeasibility. Note that $M^{v\text {in}}_{u_i\text {in}}(0)$, $M^{v\text {out}}_{u_i\text {in}}(0)$ are undefined and $M^{v\text {out}}_{u_i\text {out}}(0)=M^{v\text {in}}_{u_i\text {out}}(0)=0$. Now we calculate $M^{v'\text {in}}_{v\text {in}}(\ell )$. Observe that $M^{v'\text {in}}_{v\text {in}}(\ell )$ is equal to the optimal value of the following knapsack problem $(IP_2)$ (denoted Knapsack$_v(\ell )$) plus $\omega _v$:If $M^{v\text {out}}_{u_i\text {in}}$(s) and $M^{v\text {out}}_{u_i\text {out}}(s)$ do not have finite values then they are removed from Knapsack$_v(\ell )$. Note that $x_{i0}\equiv 0$, for any $i\in [n_v]$. If Knapsack$_v(\ell )$ has a feasible solution, then we get the value $M^{v'\text {in}}_{v\text {in}}(\ell )$, otherwise we set $M^{v'\text {in}}_{v\text {in}}(\ell )$ to be undefined. Similarly we calculate $M^{v'\text {in}}_{v\text {out}}(\ell )$, $M^{v'\text {out}}_{v\text {in}}(\ell )$ and $M^{v'\text {out}}_{v\text {out}}(\ell )$, $\ell =0,1,\ldots ,p$. From the analysis of the dynamic programming approach without global constraints, we know that if there is an FPTAS for Knapsack$_v(\ell )$, then there is FPTAS for Knapsack$_{root}(p)$ giving an FPTAS for PG with binary variables on directed trees. Note that the constraint $\sum _{s=0}^{p}(x_{is}+y_{is})=1$ can be replaced by $\sum _{s=1}^{p}(x_{is}+y_{is})\le 1$, $\forall i \in [n_v]$. By Proposition 4 we have the following:

##### Theorem 6

There is a truthful in expectation mechanism for PG with binary variables on directed trees, which is an FPTAS.

For general $x_v\in \mathbb {Z}$, we can replace each $x_v$ by $q_v$ duplicated variables $x_{vj}$, $j=1,\ldots ,q_v$, i.e., $\{x_v \in \{0,1,\ldots , q_v\}\}=\{\sum _{j\in [q_v]}jx_{vj}\,|\, \sum _{j\in [q_v]}x_{vj}\le 1, x_{vj}\in \{0,1\}\}$. Note that this transforms a polynomial size integer constraint into a multiple choice, one dimensional knapsack constraint. Hence, for directed trees, by a DP approach, we can construct a pseudo polynomial time algorithm to compute the exact optimal value of PG with integer variables, in time $poly(|V|,q,OPT^{in}(PG))$. In addition, we can remove $OPT^{in}(PG)$ from the running time by a loss of $\epsilon$ of the optimal value using scaling techniques. Thus, there is a $(1-\epsilon )$-approximation algorithm for PG with integer variables with running time in $poly(|V|,q,1/\epsilon )$. Finally, by Proposition 4, we obtain the following:

##### Theorem 7

There is a truthful in expectation mechanism for PG with polynomial size integer variables on directed trees, which is an FPTAS.

### Deterministic Truthful Mechanisms on Directed Trees

We will use a maximal in range (MIR) mechanism, see, e.g., [17], to obtain a $(3+\epsilon )$ approximate deterministic truthful mechanism for PG with polynomial size integer variables on directed trees. As we know, by transformation from integer constraint into multiple choice and one dimensional knapsack constraint, we only need to show such an approximation algorithm for binary variables. Our mechanism is based on a recent deterministic truthful PTAS for 2 dimensional knapsack problem^4^ [13, 17, 35]. We will first need the following:

#### Definition 1

A vertex in a rooted directed tree is called at level *i* if the distance between the vertex and the root is *i* in the undirected version of the tree.

Let $L_i$ denote the set of vertices of level $3k+i$, $k=0,1,2,...$, for any $i\in \{1,2,3\}$. For each vertex *v*, suppose that the number of children of *v* is $n_v$, and that the children are $u_1$, $u_2$, $\ldots$, $u_{n_v}$. Recall that $\Delta =\max _{v\in V}\{n_v\}+1$. Let $G_v$ denote the subtree constructed by *v* and its children. Then restricting PG on $G_v$ with capacity (global constraint) constraint $c_v$ and $x_v=0$ is equivalent to solving the following linear programming problem (denoted as $PG_v$):where $c_{u_iv}=1$, for $i\in [n_v]$. For any solution $s_v$ of $PG_v$, we use $\omega (s_v)$ to denote the objective value of this solution given the input $I_v=(c,w,p_v,c_v,\omega )$ and $OPT(PG_v(c_v))$ to denote the optimal value of $PG_v(c_v)$ given input $I_v$.

#### Lemma 2

[13, 35] There exists a range ${\mathcal {S}}_v(c_v)$ of solutions of $PG_v(c_v)$, which does not depend on the declarations of $I_v$ and only depends on $c_v$ such that

$$\begin{aligned} \max _{s_v\in {\mathcal {S}}_v(c_v)}\{\omega (s_v)\}\ge (1-\epsilon )OPT(PG_v(c_v)) \end{aligned}$$

Besides, there exists an $O(\Delta ^{4+\frac{1}{\epsilon }})$ algorithm ${\mathcal {A}}_v(c_v)$ that finds the optimal solution of the range ${\mathcal {S}}_v(c_v)$, for any $\epsilon >0$.

Denote now by $PG_i$ the restriction of PG on $L_i$ and let

$$\begin{aligned} {\mathcal {S}}_i=\bigcup _{v\in L_i,c_v\in [n_v],\sum _{v\in L_i}c_v\le p}{\mathcal {S}}_v(c_v) \end{aligned}$$

Then ${\mathcal {S}}_i$ is a range of $PG_i$, $i\in \{1,2,3\}$.

#### Lemma 3

1. $$\max _{s_i\in {\mathcal {S}}_i}\{\omega (s_i)\}\ge (1-\epsilon )OPT(PG_i)$$.
2. There exists an $O(|L_i|\Delta ^{6+\frac{1}{\epsilon }})$ algorithm ${\mathcal {A}}_i$ that finds the optimal solution of the range ${\mathcal {S}}_i$ of $PG_i$, for any $\epsilon >0$.

#### Proof

Suppose that in the optimal solution of $PG_i$, each vertex $PG_v$ is allocated $c^*_v$ amount of global pollution level. As we know $\sum _{v\in L_i} c^*_v\le p$. Then

$$\begin{aligned} \max _{s_i\in {\mathcal {S}}_i}\{\omega (s_i)\}\ge & {} \sum _{v\in L_i}\max _{s_v\in {\mathcal {S}}_v(c^*_v)}(\omega (s_v))\ge (1-\epsilon ) \sum _{v\in L_i} OPT(PG_v(c^*_v))\\= & {} (1-\epsilon )OPT(PG_i) \end{aligned}$$

where the first inequality comes from $\sum _{v\in L_i}c^*_v\le p_v$, the second one is due to Lemma 2, and the third one is from the definition. Suppose the fathers of vertices in $L_i$ are labeled as $v_1, v_2, \ldots , v_{\ell _i}$. Let $g_{i}(C)$ denote the optimal value of PG restricted to vertices with fathers $v_1, v_2,\ldots , v_i$ on the range ${\mathcal {S}}_i$ when the capacity allocated to this subproblem is no more than *C*. We have the following recursive function:

$$\begin{aligned} g_{i+1}(C)=\max _{c_{v_{i+1}}\le C}\{g_i(C-c_{v_{i+1}})+OPT(PG_{v_{i+1}}(c_{v_{i+1}}))\} \end{aligned}$$

where $OPT(PG_i)=\max _{i\in [\ell _{i}],C\le p}g_{i}(C)$. The total running time of this dynamic programming approach is $O(|L_i|\Delta ^{2}\Delta ^{4+\frac{1}{\epsilon }})=O(|L_i|\Delta ^{6+\frac{1}{\epsilon }})$. $\square$

#### Theorem 8

There is a deterministic $\rho ^{in}$-approximate truthful mechanism for PG with polynomial size integer variables on directed trees, where $\rho ^{in}=3+\epsilon$. For binary variables the mechanism terminates in $O(|V|^2\Delta ^{6+\frac{1}{\epsilon }})$ time.

#### Proof

We only need to prove this theorem for PG with binary variables. Note that $\max _{i\in \{1,2,3\}}\{OPT(PG_i)\}\ge \frac{1}{3}OPT(PG)$, then by Lemma 3 we have

$$\begin{aligned} \max _{i\in \{1,2,3\}}\{\max _{s_i\in {\mathcal {S}}_i}\{\omega (s_i)\}\}\ge \frac{1-\epsilon }{3}OPT(PG) \end{aligned}$$

Using the VCG payment rule within VCG mechanisms, see [17], on the range ${\mathcal {S}}=\bigcup _{i\in \{1,2,3\}}{\mathcal {S}}_i$, we can get a deterministic truthful mechanism for PG on directed trees, achieving $\frac{1-\epsilon }{3}OPT(PG)$ social welfare. The running time $O(|V|^2\Delta ^{6+\frac{1}{\epsilon }})$ follows directly by Lemma 3 and the payment rule of VCG. $\square$

## Planar Graphs

We present two algorithms for PG on planar graphs. The first has a constant approximation ratio, obtained by decomposing the plane and not violating any constraint. The second algorithm is a PTAS, obtained by a rounding of variables and a dynamic programming approach on a tree decomposition and violating the local constraints by a small value $\delta > 0$.

### Constant Approximation Without Violations

**Fig. 4:**
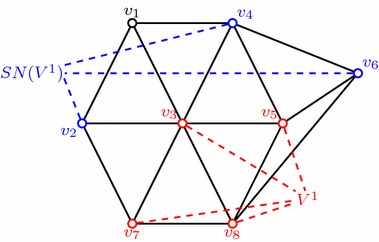
Significant neighbours $SN(V^1)=\{{v_2,v_4,v_6} {\}}$ of $V^1=\{{v_3,v_5,v_7,v_8 }{\}}$ in the graph of solid black lines

Given a digraph $G=(V,E)$ and a subset $U\subset V$, we call significant neighbours of *U*, $SN_G(U)$, all the vertices in $V \backslash U$ with at least two neighbours in *U* (see Fig. 4). Consider a partition $\{ V^i\}^\alpha _{i=1}$ of *V*. Now let ${SN}_{G^{un}}(V^i)=\{u\notin V^i\,|\, \exists v\in V^i,\, \text {s.t.}\, u\,$ is a significant neighbour of $\, v\, \text {w.r.t.}\, V^i \}$ denote the significant neighbours of $V^i$ in $G^{un}$. Let $G^i$ be the induced subgraph of $V^i\cup {SN}_{G^{un}}(V^i)$ in $G^{un}$. A partition $\{ V^i\}^\alpha _{i=1}$ of *V* is called an $(\alpha , \beta )$-partition (or $(\alpha , \beta )$-decomposition) of *G* if for any $i \in [\alpha ]$ and $v \in V^i, |\delta _{G^i}(v)| \le \beta$, where $\alpha ,\beta$ are two given positive integers.

According to the following Lemma 4, we can obtain a constant-factor approximation for PG with integer variables for any graph with $(\alpha ,\beta )$-decomposition. Such a decomposition of planar graphs will be presented later.

#### Lemma 4

If a directed graph *G* has an $(\alpha ,\beta )$-decomposition, then there is a deterministic $(\rho ^{fr}=\alpha \gamma _{\beta +2}+1)$-approximation algorithm for PG with integer variables, and, a truthful in expectation mechanism for the same problem with the same approximation.

#### Proof

If there is an $\rho ^{fr}$-approximation algorithm for a linear packing problem with binary variables, then there is an $(\rho ^{fr}+1)$-approximation algorithm for the same problem with integer variables [10]. Hence, it is sufficient to show that there is an $(\rho ^{fr}=\alpha \gamma _{\beta +2})$-approximation algorithm for PG with binary variables. So, consider a PG with binary variables. Let $\{V^i\}_{i=1}^{\alpha }$ be an $(\alpha ,\beta )$-decomposition of graph *G*. Let $x^*$ be the optimal fractional solution of PG with binary variables. Then $R(x^*)\le \alpha \max _{i\in [\alpha ]}\{R(x^*_{V^i})\}$, where $x^*_{V^i}$ is a fractional solution such that its value is equal to $x^*_v$, for any $v\in V^i$ and 0 otherwise. Let $PG_i$ denote the PG on *G* by setting $x_v=0$, for any $v\notin V^i$. Note that $x^*_{V^i}$ is a feasible solution for $PG_i$, which gives $R(x^*_{V^i})\le OPT^{fr}(PG_i)$. Without loss of generality, we suppose $w_{uv}\le p_v$, for any $(u,v)\in E$ and $v\in V$ (otherwise $x_v\equiv 0$ for PG). Observe that in $PG_i$, only $x_v$, $v\in V^i$ are variables. Now for any $v\in V^i$, let us see how many constraints in $PG_i$ contain $x_v$. Suppose $u\in V\backslash V^i$ is a neighbour of *v* in $G^{un}$. If *u* is not a significant neighbour of *v*, since $w_{vu}\le p_u$, we can remove the constraint $w_{vu}x_v\le p_v$ in $PG_i$. Hence, only the local constraints of the significant neighbours of *v* remain containing variable $x_v$. As $\{V^i\}_{i=1}^{\alpha }$ is an $(\alpha ,\beta )$-decomposition of graph *G*, there are at most $\beta +1$ local constraints containing variable $x_v$ (which includes the local constraint of vertex *v* itself). Together with the global constraint, we know $x_v$ appears in at most $\beta +2$ constraints in $PG_i$, for any $v\in V^i$, which means $PG_i$ is $\beta +2$ column sparse. Therefore, by Proposition 1, there is a polynomial deterministic algorithm for $PG_i$ with binary variables, finding an integer solution $y_i$ for $PG_i$ such that $\gamma _{\beta +2}R(y_i)\ge OPT^{fr}(PG_i), \text { for any } i\in [\alpha ]$. Then

$$\begin{aligned} \alpha \gamma _{\beta +2}\max _{i\in [\alpha ]}\{R(y_i)\}\ge & {} \alpha \max _{i\in [\alpha ]}\{OPT^{fr}(PG_i)\}\ge \alpha \max _{i\in [\alpha ]}\{R(x^*_{V^i})\} \ge R(x^*)\\= & {} OPT^{fr}(PG) \end{aligned}$$

A truthful in expectation mechanism with the same approximation ratio is guaranteed by Proposition 3. $\square$

*Planar graphs* In the following, we will show that the integrality gap of PG on planar graphs is at least 4 as shown by a complete graph with four vertices. For a small $\epsilon >0$, let $w_{uv}=\epsilon$, for any $(u,v)\in E$, and $p_v=\omega _{v}=1$, for any $v\in V$. There is no global constraint. The optimal integer solution of PG on this graph is $x_v=1$ for some $v\in V$ and $x_u=0$ for all $u\ne v$, implying the optimal objective value 1. However, setting $x_v=1-4\epsilon$, for any $v\in V$ provides a feasible fractional solution, which gives the objective value $4-16\epsilon$. Therefore, the integrality gap is at least 4, meaning that our LP relaxation cannot lead to better than 4 (e.g., PTAS) approximations.

We provide an $(\alpha ,\beta )$-decomposition of any planar graph, with $\alpha =18$, $\beta =6$. We did not attempt to optimize these two parameters.

#### Theorem 9

There is an $(\alpha ,\beta )$-decomposition of a directed planar graph $G=(V,E)$, where $(\alpha ,\beta )=(18,6)$.

#### Proof

Let $G'=G^{un} = (V,E')$. Suppose $G'$ is connected, otherwise we can run the algorithm on each connected component separately. Define a sequence of vertex sets $\{N_i\}_i$ of $G'$ as follows in a BFS manner. Fix an arbitrary vertex $v_0\in V$, and let $N_1=\{v_0\}$, and $N_i$ is defined recursively as

$$\begin{aligned} N_{i+1}=\left\{ v\in V\backslash \bigcup _{j=1}^{i}N_{j}\,|\, (v,u)\in G',\, \text {for some}\, u\in N_{i} \right\} , \end{aligned}$$

for $i=1,2,\ldots ,|V|$. By this definition, for any $v\in N_i$ and $u\in N_j$, if $|i-j|\ge 2$, then $(u, v) \notin E'$. We also observe that $N_i$ is the set of vertices with distance $i-1$ to $v_0$ in $G'$ (i.e., the shortest path distance with respect to the number of edges). Suppose the length of the sequence $\{N_i\}_i$ is *K*. Let $S_i=\{j\equiv i\,(\text {mod}\, 3)\,|\,j\in [K]\}$, $i\in \{1,2,3\}$. Let $S_0=S_3$, and $V^i=\bigcup _{j\in S_i}N_j$, $i\in \{1,2,3\}$. We will need the following Lemmas 5, 6 and 7.

#### Lemma 5

For each $v\in N_j$, the number of significant neighbours of *v* in $N_{j-1}$ with respect to $N_j$ is at most two.

#### Proof

**Fig. 5:**
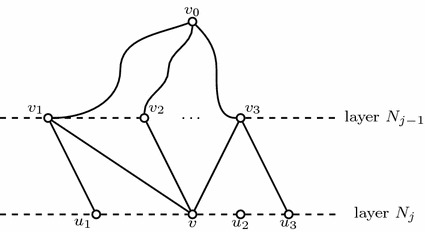
An illustration of relations between $N_j$ and $N_{j-1}$

Suppose towards a contradiction that *v* has three significant neighbours with respect to $N_j$. That is, suppose there exists $v_1\ne v_2 \ne v_3 \in N_{j-1}\cap \delta _{G'}(v)$ and $v\ne u_1,u_2,u_3 \in N_j$ such that $(u_i,v_i)\in G'$, $i\in \{1,2,3\}$ (see Fig. 5). By the definition of $N_j$, there is a path from $v_0$ to $v_i$, $i\in \{1,2,3\}$, and $(v,v_i)\in G'$, $i\in \{1,2,3\}$. Suppose without loss of generality that $v_2$ is inside the circle constructed from the path of $v_0$ to $v_1$, $v_3$ and edges $(v,v_1)$ and $(v,v_3)$ in the planar embedding. Then $(u_2,v_2)$ will intersect this circle, which contradicts that $G'$ is planar. $\square$

Next, we partition $N_j$ into two sets $N^1_j$ and $N^2_j$ such that each vertex in $N^i_j$ has at most two significant neighbours in $N_{j+1}$ with respect to $N^i_j$, $i\in \{1,2\}$. We say two vertices $v, u \in N_j$ are connected by a zigzag path if there exists a path $(v,v_1,v_2,v_3,\ldots ,v_s,u)$ in $G'$ such that $v_{i}$ and $v_{i+1}$ alternatively belong to $N_{j+1}$ and $N_{j}$, i.e., $v_1\in N_{j+1}$ and $v_2\in N_j$. Note that *s* must be odd. We define the zigzag length of this zigzag path as $\frac{s+1}{2}$. The zigzag distance between *v* and *u*, denoted $d^{z}_{uv}$, is defined as the zigzag length of the shortest zigzag path between *v* and *u* if there exists one and $\infty$ otherwise. Note that the zigzag distance of *v* to itself is zero. The partition algorithm PA$_1$ (see Algorithm 1) partitions the set of vertices in layer $N_j$ into sets $N_j^1$ and $N_j^2$. The algorithm proceeds in iterations. In each iteration a vertex $v \in N_j$ is chosen arbitrarily and all vertices with odd zigzag distance from *v* are assigned to $N_j^1$. Similarly all vertices with even zigzag distance are assigned to $N_j^2$. Let $N^1_j=A_1$ and $N^2_j=A_2$, where $A_1, A_2$ is the output of PA$_1$. (Note that PA$_1$ runs for each $j \in [K]$.)

Intuitions behind $A_1, A_2$ are quite simple. We want to avoid more than two significant neighbours for a vertex $v \in N_j$ that reside in $N_{j+1}$. The only way to have a significant neighbour of *v* in $N_{j+1}$ is to have a single zigzag from *v* to a vertex in $N_{j+1}$ and back to a vertex in $N_j$. Thus it is enough to put two consecutive vertices from $N_j$ joined by two such consecutive zigzags, into the same set of the partition. And $A_1$ and $A_2$ are precisely those vertices from $N_j$ which lie on odd ($A_1$) and even ($A_2$) zigzags, respectively, and thus such a partition does the job.
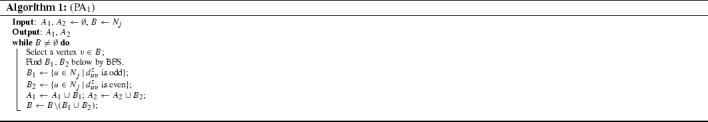


#### Lemma 6

For each $v\in N^i_j$, *v* has at most two significant neighbours in $N_{j+1}$ with respect to $N^i_j$, $i\in \{1,2\}$.

#### Proof

**Fig. 6:**
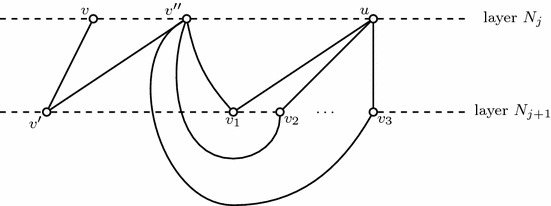
Relations between $N_j$ and $N_{j+1}$

First, note that if *v* and *u* are selected in different iterations of the while loop in Algorithm 1, there is no zigzag path between them. Therefore, for a single iteration of the while loop, suppose $v\in B$ is selected. We only need to show that for any $u\in B_i$, *u* has at most two significant neighbours in $N_{j+1}$ with respect to $B_i$, $i\in \{1,2\}$. First, note that $v\in B_2$ (its zigzag distance to itself is 0). Since all the other vertices in $B_2$ have zigzag distance to *v* at least two, *v* has no significant neighbours with respect to $B_2$ in $N_{j+1}$. Now fix $i\in \{1,2\}$. Consider any two vertices $u, u' \in B_i$, where *u* and $u'$ connect to the same vertex in $N_{j+1}$ only if they have the same zigzag distance to *v*. Suppose there exist three different vertices $v_1$, $v_2$, $v_3 \in N_{j+1}$, such that they are significant neighbours of *u* with respect to $B_i$ (see Fig. 6). By similar arguments as above, there exists zigzag paths from *v* to $v_i$, $i\in \{1,2,3\}$. Also note that edges $(u,v_i)\in G', i\in \{1,2,3\}$. Without loss of generality, suppose $v_2$ is in the circle constructed from the zigzag paths *v* to $v_1$, $v_3$ and edges $(u,v_1)$ and $(u,v_3)$. Since $G'$ is planar, there exists no edge between $v_2$ and another vertex in $B_i$ with the same zigzag distance to *u*. Therefore, *u* has at most two significant neighbours with respect to $B_i$ in $N_{j+1}$. $\square$

Next we will partition each set $N^i_{j}$, $i\in \{1,2\}$, $j\in [K]$ into a constant number of sets $\{N^{ik}_j\}_k$ such that each vertex in $N^{ik}_j$ has at most a constant number of significant neighbours with respect to $N^{ik}_j$ in $N_{j}$. We provide two partition algorithms namely PA$_2$ and PA$_3$. Both algorithms are similar in spirit to Algorithm 1. For any two vertices *v*, $u\in N^i_j$, we say that they are connected by an $N_j$-path if there exists a path $(v,v_1,v_2,\ldots ,v_s,u)$ in $G'$ such that $v_{\ell }\in N_j$, $\forall \ell \in [s]$. $N_j$-distance of two vertices *v*, $u\in N^i_j$, denoted $d^{N_j}_{uv}$, is defined as the number of edges of the shortest $N_j$-path between *v* and *u* if there exists one and $\infty$ otherwise. PA$_2$ (Algorithm 2) partitions the vertices of each $N_j^i$ into three sets $N_j^{ik}, k \in \{1,2,3\}$ in such way that $N_j^{ik}$ contains the vertices with $N_j$-distance from an arbitrarily chosen vertex $v \in N_j^i$, in every iteration of the algorithm. Note that $v \in B_3$, because the $N_j$-distance from *v* to itself is zero. Let $N^{ik}_j=A_k$, $k\in \{1,2,3\}$, where $A_1$, $A_2$, $A_3$ are a partition of $N^i_j$ output by PA$_2$.
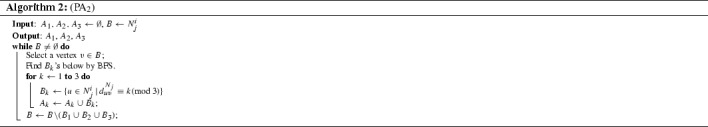


#### Lemma 7

For any $k\in \{1,2,3\}$, and each $v\in N^{ik}_j$, *v* has at most 2 neighbours in $N_j$, or has no neighbours in $N^{ik}_j$ nor significant neighbours with respect to $N^{ik}_j$ in $N_j\backslash N^{ik}_j$.

#### Proof

**Fig. 7:**
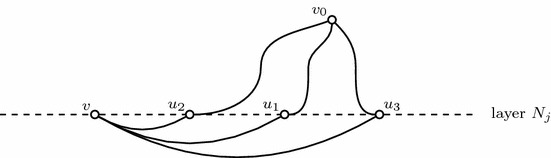
Relations between $N_j$ and $N_{j}$

First, note that if *v* and *u* are selected in different iterations of while loop in Algorithm 2, there is no $N_j$-path between them. Therefore, for a single iteration of the while loop, suppose $v\in B$ is selected. Since $v\in B_3$ ($N_j$ distance to itself is 0), *v* has no neighbours in $B_3$ nor significant neighbours with respect to $B_3$ in $N_j\backslash B_3$ by PA$_2$. Now fix $k\in \{1,2,3\}$. Consider any two vertices $u_1, u_2 \in B_k$, $u_1$ and $u_2$ connect to the same vertex in $N_j$ only if they have the same $N_j$-distance to *v*. Next we will show for any $u_1\ne v$ and $u_1\in B_k$, for any *k*, $u_1$ has at most two neighbours in $N_j$. Suppose there exist three different vertices $u_1$, $u_2$, $u_3 \in N_j$, such that $(u_1,u_2)\in G'$ and $(u_1,u_3)\in G'$. By similar arguments as above, there exists $N_j$-paths from *v* to $u_i$, $k\in \{1,2,3\}$. Since $u_i \in N_j$, $i\in \{1,2,3\}$, there exist paths in $G'$ from $v_0$ to $u_i$, $i\in \{1,2,3\}$. We observe that it is only possible that $u_1$ is in the circle constructed from the $N_j$ paths *v* to $u_2$, $u_3$ and paths from $v_0$ to $u_2$ and $u_3$ (the case where $u_2$ or $u_3$ is in the circle constructed by the other two vertices with *v* and $v_0$ will violate the planarity of $G'$) (see Fig. 7). Since graph $G'$ is planar, there exists no edge between $u_1$ and another vertex in $N_j$ (due to that such a vertex will have a path to *v* and $v_0$ respectively). Therefore, $u_1$ has at most two neighbours in $N_j$. $\square$

Combining Lemmas 5, 6 and 7, $\{N^{ik}_j\}_{ijk}$ is an $(\alpha ,\beta )$-decomposition of *G* with $(\alpha ,\beta )=(18,6)$, which finishes the proof of Theorem 9. $\square$

By Theorem 9 and Lemma 4, we have

#### Theorem 10

There is a randomized, individually rational and truthful in expectation $(18\gamma _8+1)$-approximation mechanism for PG on planar graphs with integer variables.

### Better Approximation Under Some Mild Condition

We will use the 4-color theorem for planar graphs to present an improved $(6+\epsilon )$-approximate truthful in expectation mechanism for PG under the following natural (and mild) assumption:

12 $$\begin{aligned} \sum _{u\in \delta ^{-}_G(v)}w_{uv}\le p_v \end{aligned}$$

This constraint means that if each of *v*’s neighbours emits only one unit amount of pollution, the level of pollution in *v* will not exceed *v*’s local level of pollution. Let $x^1$ be the optimal fractional solution of PG with binary variables without global constraint on planar graph *G*.

#### Theorem 11

Suppose condition (12) holds and $R(x^1)\ge 1$. There is a randomized, individually rational, $(\rho ^{fr}=6+\epsilon )$-approximation mechanism that is truthful in expectation for PG on planar graphs with integer variables, terminating in time $poly(|I|,\log (\frac{1}{\epsilon }))$.

#### Proof

Note that if condition (12) holds, then every independent set is a feasible solution for PG with binary variables without global constraint. By 4-color theorem [7, 46] for planar graphs, there is an independent set $S\subset V$ such that $4R(z_S)\ge R(x^1)$ where $z_S$ is defined by $z_v=1$ if $v\in S$ and $z_v=0$ otherwise. Further there is an $O(|V|^2)$ algorithm finding $z_S$ [46]. By Theorem 3 of [36] and $R(x^1)\ge 1$, there is a deterministic $(\rho ^{fr}=5+\epsilon )$-approximation algorithm for PG with binary variables, running in $poly(|I|,\log (\frac{1}{\epsilon }))$ time. Then there is a deterministic $(\rho ^{fr}=6+\epsilon )$-approximation algorithm for PG with integer variables, running in time $poly(|I|,\log (\frac{1}{\epsilon }))$ [10]. By Proposition 3, this $(\rho ^{fr}=6+\epsilon )$-approximation mechanism is truthful in expectation for PG with integer variables. $\square$

### A PTAS with $\delta$ Violation of Constraints

*A PTAS with*$\delta$*-violation* Our approach to obtain a PTAS has three main steps:

1. Round *PG* to an equivalent problem ${\bar{PG}}_2$ with polynomial size integer variables.
2. Using the nice tree decomposition [34], we present a dynamic programming approach to solve ${\bar{PG}}_2$ optimally on a *k*-outerplanar graph.
3. By a shifting technique similar to [8], we obtain a PTAS with $1+\delta$ violation of local constraints for *PG*.

*Step 1: Rounding Procedure*

Recall that PG is equivalent to the following integer linear program:where $$\omega _v=\max \{0, s^0_v-s^1_v-\sum _{u\in \delta ^{+}_G(v)}s^1_uw_{vu}\}$$ and $$w_{v,v}=1$$$$\forall v\in V$$, and $$b_v$$ and $$d_v$$ are both linear with slopes $$s^0_v$$ and $$s^1_v$$. For each $$v\in V$$, suppose $$q_v\in [2^{o_v-1}-1, 2^{o_v}-1)$$. Let $$o_v=\lfloor \log _2(q_v)\rfloor +1$$ if $$q_v\ne 2^{o_v-1}-1$$ and $$o_v=\lfloor \log _2(q_v)\rfloor +2$$ otherwise; $$c^i_v=2^{i-1}$$, $$i\in [o_v-1]$$ and $$c^{o_v}_v=q_v-2^{o_v-1}+1$$. By simple calculations, we know

$$\begin{aligned} \{x_v\,|\, x_v\in \mathbb {Z}, 0\le x_v \le q_v\}=\left\{ \sum _{i=1}^{o_v}c^i_vy^i_v\,|\, y^i_v\in \{0,1\}, i\in [o_v]\right\} \end{aligned}$$

for any $v\in V$. Therefore, PG is equivalent to the following integer linear programming problem (denoted as $PG'$):Let $o^*=\max _{v\in V}o_v$ and $\rho =o^*|V|$. Recall that $q=\max _{v\in V}\{q_v\}+1$. For any $\delta >0$, let

$$\begin{aligned} {\bar{w}}^i_{uv}=\left\lfloor \frac{2w_{uv}c^i_v\rho }{p_v\delta }\right\rfloor \text { and } {\bar{p}}_v=\left\lceil \frac{2p_v\rho }{p_v\delta }\right\rceil =\left\lceil \frac{2\rho }{\delta }\right\rceil \end{aligned}$$

for any $u,v\in V$. Then we have the following modified $PG'$ denoted as ${\bar{PG}}_1$:

#### Lemma 8

Any feasible solution of $PG'$ is feasible in ${\bar{PG}}_1$, and any feasible solution of ${\bar{PG}}_1$ is feasible for PG except violating each local constraint by a factor of $1+\delta$.

#### Proof

We only prove local constraints for each direction since the proof of the global constraint is similar. Let $\{y^i_v\}_{v\in V,\, i\in [o_v]}$ be a feasible solution of $PG'$. We know that

$$\begin{aligned} \sum _{i=1}^{o_v}w_{vv}c^i_vy^i_v+\sum _{u\in \delta ^{-}_G(v)}\sum _{i=1}^{o_v}w_{uv}c^i_uy^i_v \le p_v, \forall v \in V \end{aligned}$$

Then

$$\begin{aligned} \sum _{i=1}^{o_v}{\bar{w}}^i_{vv}y^i_v+\sum _{u\in \delta ^{-}_G(v)}\sum _{i=1}^{o_v}{\bar{w}}^i_{uv}y^i_v\le & {} \frac{2\rho }{p_v\delta }\Bigg (\sum _{i=1}^{o_v}w_{vv}c^i_vy^i_v+\sum _{u\in \delta ^{-}_G(v)}\sum _{i=1}^{o_v}w_{uv}c^i_uy^i_v\Bigg )\\\le & {} \frac{2\rho }{p_v\delta } p_v\le {\bar{p}}_v \end{aligned}$$

as desired. On the other hand, suppose $\{y^i_v\}_{v\in V,\, i\in [o_v]}$ is a feasible solution of ${\bar{PG}}_1$. We know that

$$\begin{aligned} \sum _{i=1}^{o_v}{\bar{w}}^i_{vv}y^i_v+\sum _{u\in \delta ^{-}_G(v)}\sum _{i=1}^{o_v}{\bar{w}}^i_{uv}y^i_v \le {\bar{p}}_v, \forall v \in V \end{aligned}$$

Then

$$\begin{aligned} \begin{aligned}&\sum _{i=1}^{o_v}w_{vv}c^i_vy^i_v+ \sum _{u\in \delta ^{-}_G(v)}\sum _{i=1}^{o_v}w_{uv}c^i_uy^i_v\\&\quad \le \frac{p_v\delta }{2\rho } \Bigg ( \sum _{i=1}^{o_v}({\bar{w}}^i_{vv}+1)y^i_v+ \sum _{u\in \delta ^{-}_G(v)} \sum _{i=1}^{o_v}({\bar{w}}^i_{uv}+1)y^i_v \Bigg )\\&\quad \le \frac{p_v\delta }{2\rho } \sum _{i=1}^{o_v}{\bar{w}}^i_{vv}y^i_v+\sum _{u\in \delta ^{-}_G(v)}\sum _{i=1}^{o_v}{\bar{w}}^i_{uv}y^i_v+\frac{p_v\delta \rho }{2\rho }\\&\quad \le \frac{p_v\delta {\bar{p}}_v}{2\rho }+\frac{p_v\delta }{2} \le \frac{p_v\delta }{2\rho }\bigg (\frac{2\rho }{\delta }+1\bigg ) +\frac{p_v\delta }{2}\\&\quad \le p_v(1+\delta ), \forall v\in V \end{aligned} \end{aligned}$$

$$\square $$

Note that for each $\ell \in [q_v]$, there is a solution $\{y^i_v\}_{i\in [o_v]}$ such that $\sum _{i=1}^{o_v}c^i_vy^i_v=\ell$. If $\ell \le 2^{o_v-1}-1$, set $y^{o_v}_v=0$ and if $2^{o_v-1}-1<\ell \le q_v$, set $y^{o_v}_v=q_v-2^{o_v-1}+1$. In both cases there is a unique solution such that $\sum _{i=1}^{o_v}c^i_vy^i_v=\ell$. Hence, there is a one-to-one correspondence from $x_v$ to $\{y^i_v\}_{i\in [o_v]}$. It is not difficult to see that for a given $x_v$, the solution $\{y^i_v\}_{i\in [o_v]}$ defined above is the one such that $\sum _{i=1}^{o_v}{\bar{w}}^i_{vv}y^i_v+\sum _{u\in \delta ^{-}_G(v)}\sum _{i=1}^{o_v}{\bar{w}}^i_{uv}y^i_v$ is minimized. Now let ${\bar{w}}_{vu}(x_v)=\sum _{i=1}^{o_v}{\bar{w}}^i_{vu}y^i_v$, for any $v, u \in V$, where $\{y^i_v\}_{i\in [o_v]}$ corresponds to the solution of $x_v$. Let $\varLambda _v=[q_v]\cup \{0\}$. Using these notations, we know that ${\bar{PG}}_1$ (also PG) is equivalent to the following integer linear programming problem (denoted as ${\bar{PG}}_2$):*Step 2: Preliminaries of tree decompositions onk*-*outerplanar graphs*

#### Definition 2

A tree decomposition of an undirected graph $G=(V,E)$ is a pair $(\{X_i|i\in I\},\,T=(I,F))$, with $\{X_i|i\in I\}$ a family of subsets of *V*, one for each node of *T*, and *T* a tree such that:

1. $$\bigcup _{i\in I}X_i=V$$,
2. for all edges $(v,w)\in E$, there exists an $i\in I$ with $v\in X_i$ and $w\in X_i$,
3. for all *i*, *j*, *k*$\in I$: if *j* is on the path from *i* to *k* in *T*, then $X_i\cap X_k\subseteq X_j$.

The width of a tree decomposition $(\{X_i|i\in I\},\,T=(I,F))$ is $\max _{i\in I}|X_i|-1$. The minimum width of all tree decompositions of *G* is called treewidth.

#### Definition 3

A tree decomposition $(\{X_i|i\in I\},\,T=(I,F))$ of $G=(V,E)$ is called a nice tree decomposition if *T* is a rooted binary tree and

1. if a node $i\in I$ has two children *j* and *k*, then $X_i=X_{j}=X_{k}$ (joint node),
2. if a node $$i \in I$$ has one child *j*, then either $$X_i\subset X_j$$, and $$|X_i| = |X_j| - 1$$ (forget node), or $$X_j \subset X_i$$ and $$|X_j| =$$$$|X_i| - 1$$ (introduce node),
3. if node $i \in I$ is a leaf of *T*, then $|X_i| = 1$ (leaf node).

#### Lemma 9

[29] For any *k*-outerplanar graph $G=(V,E)$, there is an algorithm to compute a tree decomposition $(\{X_i|i\in I\},\,T=(I,F))$ of *G* with treewidth at most $3k-1=O(k)$, and $I=O(|V|)$ in *O*(*k*|*V*|) time.

Given a tree decomposition $(\{X_i|i\in I\},\,T=(I,F))$ for $G=(V,E)$ with treewidth *k* and $I=O(|V|)$, we can obtain a nice tree decomposition with the same treewidth *k* and the same number of nodes *O*(*k*|*V*|) in $O(k^2|V|)$ time [34]. Thus, for any *k*-outerplanar graph $G=(V,E)$, we can compute a nice tree decomposition $(\{X_i|i\in I\},\,T=(I,F))$ of *G* with treewidth at most $3k-1=O(k)$, and $I=O(k|V|)$ in $O(k^2|V|)$ time. In the following, we will assume there is a nice tree decomposition for any *k*-outerplanar graph.

*Dynamic Programming (DP)* We present a DP approach to solve ${\bar{PG}}_2$ on a directed *k*-outerplanar graph using a nice tree decomposition of its undirected version. Note that a nice tree decomposition of an undirected version of a directed graph is also a nice tree decomposition of itself. Suppose we have a nice tree decomposition $(\{X_i|i\in I\},\,T=(I,F))$ of a directed *k*-outerplanar graph $G=(V,E)$. We will use a bottom–up DP approach for ${\bar{PG}}_2$. In the following we will present our DP approach to the more general application of the allocation of pollution licences (application 2).

For any node $i\in I$, suppose $X_i=\{v^i_1,v^i_2,\ldots ,v^i_t\}$, where $t\le 3k$. We say that vertex $v^i_1$ belongs to node $X_i$. Similarly we say that a vertex belongs to a subtree of *T*, meaning that this vertex belongs to some node of this subtree. Recall that given any allocation of licences $\{x_v\}_{v\in V}$, the maximum number of cars (and so the maximum number of licences) allowed at any moment in city *v* is ${\bar{w}}_{vv}(x_v)+\sum _{u\in \delta ^{-}_G(v)} {\bar{w}}_{uv}(x_u)$ (the local constraint). Let $\mathbf {a^i}=(a^i_1,a^i_2,\ldots ,a^i_t)$ denote the number of licences allocated to vertices in $X_i$, i.e., $a^i_s$ denotes the number of licences allocated to vertex $v^i_s$, $s\in [t]$. Similarly ${\varvec{\ell }}^{\mathbf {i}}$ denotes the locally maximum number of cars allowed at any moment in vertices of $X_i$. Let $G_i$ denote the subgraph generated by all the vertices belonging to the subtree (node $X_i$) rooted at $X_i$. We use $Q^i$ to denote the total number of licences allocated to $G_i$. Let $\varPsi _i(\mathbf {a^i},{\varvec{\ell }}^{\mathbf {i}},Q^i)$ denote the optimal objective value of ${\bar{PG}}_2$ restricted to the subgraph $G_i$, when the number of licences on $v^i_s$ and the number of allowed cars at any moment on i are respectively $a^i_s$ and $\ell ^i_s$, $s\in [t]$, and the total number of licences on $G_i$ is exactly $Q^i$. If there is no feasible solution to $\varPsi _i(\mathbf {a^i},{\varvec{\ell }}^{\mathbf {i}},Q^i)$, our DP approach will automatically set $\varPsi _i(\mathbf {a^i},{\varvec{\ell }}^{\mathbf {i}},Q^i)$ to $-\infty$. Let ${\bar{w}}_{uv}(x_v)\equiv 0$ if (*u*, *v*) is not an edge in *G*. Note that the range of $a^i_s$ we need to compute is in $\varLambda _v$, $\ell ^i_s$ ranges from 0 to ${\bar{p}}_{v^i_s}$, $s\in [t]$ and $Q^i$ from 0 to *p*. The DP approach is as follows:

- $X_i$ is a leaf node or a start node, where $t=1$. $\varPsi _i(a^i_1,\ell ^i_1,Q^i)=\omega _{v^i_1}a^i_1$ if the triple $(a^i,\ell ^i,Q^i)$ is feasible, which can be verified easily e.g. $Q^i=a^i_1$ and $\ell ^i_1={\bar{w}}_{v^i_1v^i_1}(a^i_1)$. Let $\varPsi _i(a^i_1,\ell ^i_1,Q^i)=-\infty$ if the triple $(a^i,\ell ^i,Q^i)$ is not feasible.
- $X_i$ is a forget node and suppose its child is $X_j=X_i\cup \{v^j_{t+1}\}$.
$$\varPsi _i(\mathbf {a^i},{\varvec{\ell }}^{\mathbf {i}},Q^i)=\max _{a^j_{t+1},\ell ^j_{t+1}}\varPsi _j(\mathbf {a^i},a^j_{t+1},{\varvec{\ell }}^{\mathbf {i}},\ell ^j_{t+1},Q^i)$$
- $X_i$ is an introduce node and suppose its child is $X_j=X_i\backslash \{v^i_{t}\}$. Let $a^j_s=a^i_s$ and $\ell ^j_s=\ell ^i_s-{\bar{w}}_{v^i_tv^i_s}(a^i_t)$, $\forall s\in [t-1]$. $\varPsi _i(\mathbf {a^i},{\varvec{\ell }}^{\mathbf {i}},Q^i)=\varPsi _j(\mathbf {a^j},{\varvec{\ell }}^{\mathbf {j}}, Q^i-a^i_{t})+\omega _{v^{i}_t}a^i_{t}$ if $\sum _{s\in [t]}{\bar{w}}_{v^i_sv^i_{t}}(a^i_s)=\ell ^i_{t}$, and $\varPsi _i(\mathbf {a^i},{\varvec{\ell }}^{\mathbf {i}},Q^i)=-\infty$ otherwise.
- $X_i$ is a joint node and suppose its two children *j* and *k* are such that $X_j=X_k=X_i$. $\varPsi _i(\mathbf {a^i},{\varvec{\ell }}^{\mathbf {i}},Q^i)=\max _{A}\{\varPsi _j(\mathbf {a^j},{\varvec{\ell }}^{\mathbf {j}},Q^j)+\varPsi _k(\mathbf {a^k},{\varvec{\ell }}^{\mathbf {k}},Q^k)\}$, where the condition
  $$A=\{(\mathbf {a^j},{\varvec{\ell }}^{\mathbf {j}},Q^j),(\mathbf {a^k},{\varvec{\ell }}^{\mathbf {k}},Q^k)\,|\,\mathbf {a^j}+\mathbf {a^k}=\mathbf {a^i},{\varvec{\ell }}^{\mathbf {j}}+{\varvec{\ell }}^{\mathbf {k}}={\varvec{\ell }}^{\mathbf {i}}, Q^j+Q^k=Q^i\}$$.
- $X_i$ is the root of *T*, $OPT(Q^i)=\max _{\mathbf {a^i},{\varvec{\ell }}^{\mathbf {i}}}\{\varPsi _i(\mathbf {a^i},{\varvec{\ell }}^{\mathbf {i}},Q^i)\}$ is the optimal value (social welfare) of ${\bar{PG}}_2$ when the total scaled number of licences is exactly $Q^i$, i.e., the global constraint satisfies $\sum _{v\in V}x_v= Q^i$.

*Analysis of running time of DP* We next prove by induction that the above DP approach gives the optimal solution of ${\bar{PG}}_2$ on *k*-outerplanar graphs. Having a nice tree decomposition we first start from the leaf nodes computing the optimal solutions of all the nodes until we reach the root (bottom–up approach). For every node $i \in I$ we compute its optimal solution (i.e. on the subtree rooted at *i*) according to the relative case (i.e. introduce node, forget node, joint node). More formally we have the following:

#### Claim

The DP approach on the nice tree decomposition of a *k*-outerplanar graph gives the optimal solution to ${\bar{PG}}_2$.

#### Proof

We prove the claim by induction on the height *h* of the tree *T*.

*Induction base* In the case where $h=0$ the root node is the leaf hence we can easily verify whether the triple $\varPsi$ is feasible and set its value according to step 1 of the DP approach.

*Induction step* Suppose the tree has height $h>0$ rooted at node *v*. We now consider the following cases:

- *v* is an introduce node and its child is $u_1$. We assume that we have computed the optimal solution for the subtree rooted at $u_1$ (with height $h-1$). In this case the optimal value of *v* is given by the formula of the third step of the DP approach.
- *v* is a forget node and its child is $u_1$. The case is similar to the one given in the introduce node step with the difference that the optimal value of *v* is given by the formula of the second step of the DP approach.
- *v* is a joint node and its children are $u_1$ and $u_2$. We assume again that we have computed the optimal solutions for the subtrees rooted at $u_1$ and $u_2$ (where at least one has height $h-1$). Then the optimal value of *v* is given by the formula of the fourth step of the DP approach.

$$\square $$

We note that the tree is rooted at an empty forget node. Furthermore, each vertex can be introduced multiple times but can only be forgotten once. Hence, the optimal solution of the root is also the optimal solution of ${\bar{PG}}_2$ when the global constrained, $\sum _{v \in V} x_v = Q^i$, is satisfied.

For each node $X_i$, we need to keep $O(pq^{3k} \lceil \frac{2\rho }{\delta }\rceil ^{3k})=O(|V|q^{3k+1} \lceil \frac{2\rho }{\delta }\rceil ^{3k})$ number of $\varPsi _i$ values. Each $\varPsi _i$ can be computed in $O(|V|q^{3k+1} \lceil \frac{2\rho }{\delta }\rceil ^{3k})$ time (this is the worst case running time when $X_i$ is a joint node). There are *O*(*k*|*V*|) nodes in *T*. Therefore, the total running time of the DP approach (by multiplying the above three numbers) is $O(k|V|^3q^{6k+2} \lceil \frac{2\rho }{\delta }\rceil ^{6k})$.

Based on the above DP approach, we can solve ${\bar{PG}}_2$ on any *k*-outerplanar graph optimally for any fixed *k* (which includes any directed tree whose treewidth is 2). Therefore, for any $\delta >0$ and fixed *k*, we can use VCG to get an optimal deterministic truthful mechanism for PG on any directed *k*-outerplanar graph that violates each local constraint by a factor of $\delta$ and runs in $O(k|V|^3q^{6k+2} \lceil \frac{2\rho }{\delta }\rceil ^{6k})$ time (note that Theorem 12 also works for bounded treewidth graphs).

#### Theorem 12

For any $\delta >0$ and fixed *k*, there is an optimal deterministic truthful mechanism for PG on any directed *k*-outerplanar graph $G=(V,E)$ that violates each local constraint by a factor of $1+\delta$ and runs in $O(k|V|^3q^{6k+2} \lceil \frac{2\rho }{\delta }\rceil ^{6k})$ time, where $\rho =|V|(\lfloor \log _2(q)\rfloor +2)$.

*Step 3: PTAS for planar graphs*

Observe that when there are some boundary conditions on a *k*-outerplanar graph, the above DP approach still works. For example, if the number of licences of any vertex in any first and last face (level 1 and level *k* face) of the *k*-outerplanar graph is zero, we just modify the dynamic programming approach in a bottom–up manner to set $\varPsi _i=-\infty$ if any vertex *v* in any first and last face is a parameter of $\varPsi _i$ and its number of licences $a^i_v>0$. Then the modified DP approach is the desired algorithm for ${\bar{PG}}_2$ on the *k*-outerplanar graph under this boundary condition.

#### Proposition 5

PG is strongly NP-hard on planar graphs when we allow a $\delta$ violation of local constraints.

#### Proof

Suppose we restrict PG instances to fulfill that

13 $$\begin{aligned} \sum _{u\in \delta ^{-}_G(v)}w_{uv} \le p_v, \forall v \in V \end{aligned}$$

Then the maximum independent set problem can be solved as such PG problem with each $p_v=1$. Further, observe that if $\delta =\min _{u,v}\{w_{uv}\}$, then even if we allow for $(1+\delta ')$-violation of the local constraints, where $0<\delta '<\delta$, the maximum independent set problem can still be solved as such PG problem. Maximum independent set on a planar graph with degree at most 3 is strongly NP-hard [23]. $\square$

Theorem 14 provides a PTAS for PG with $q= poly(|V|)$ (in particular $q_v =1$ and 13) and $(1+\delta ')$-violation, giving a tight approximation in this sense.

#### Theorem 13

For any fixed *k* and $\delta >0$, there is an $O(k^2|V|^3q^{6k+2} \lceil \frac{2\rho }{\delta }\rceil ^{6k})$ algorithm for PG with integer variables on directed planar graph $G=(V,E)$ that achieves $\rho ^{in}$-approximation and violates each local constraint by a factor of $1+\delta$, where $\rho =|V|(\lfloor \log _2(q)\rfloor +2)$ and $\rho ^{in}=\frac{k}{k-2}$.

#### Proof

We use $$OPT({\bar{PG}}_2)$$ to denote $$OPT^{in}_G({\bar{PG}}_2)$$ omitting the superscript and subscript. By Lemma 8, we know $$OPT=OPT(PG)\le OPT({\bar{PG}}_2)$$. Let $${\bar{PG}}_2(i)$$ denote the $${\bar{PG}}_2$$ restricted on *G* when setting $$x_v=0$$ for each *v* that belongs to any face $$f\equiv i$$ or $$i+1$$$$(mod\, k)$$. Let $$\{x^*_v\}_{v\in V}$$ be an optimal solution for $${\bar{PG}}_2$$. Then we know

$$\begin{aligned} \sum _{i\in [k]}\sum _{v\in f: f\equiv i\, \text {or}\, i+1 (mod\, k)}x^*_v=2OPT({\bar{PG}}_2) \end{aligned}$$

As a consequence, there exists $i\in [k]$ such that

$$\begin{aligned} \sum _{v\in f: f\equiv i\, \text {or}\, i+1 (mod\, k)}x^*_v \le \frac{2OPT({\bar{PG}}_2)}{k} \end{aligned}$$

Observe that $$\{x_v\}_{v\in V}$$ is a feasible solution for $${\bar{PG}}_2(i)$$, where $$x_v=0$$ if *v* belongs to any face $$f\equiv i$$ or $$i+1$$$$(mod\, k)$$ and $$x_v=x^*_v$$ otherwise. Thus,

$$\begin{aligned} OPT\left( {\bar{PG}}_2\left( i\right) \right) \ge \left( 1-\frac{2}{k}\right) OPT\left( {\bar{PG}}_2\right) \ge \left( 1-\frac{2}{k}\right) OPT \end{aligned}$$

**Fig. 8:**
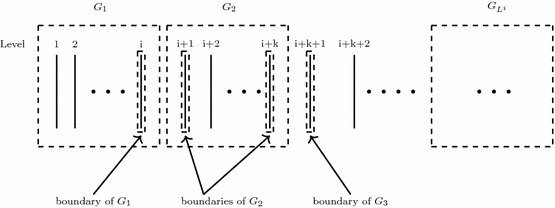
An illustration of how to select k-outerplanar graphs

Solving each $${\bar{PG}}_2(i)$$, $$i\in [k]$$, then choosing $$\max _{i\in [k]}\{OPT({\bar{PG}}_2(i))\}$$ (which is at least $$ (1-\frac{2}{k})OPT$$) gives the desired result. Now let us see how to solve $${\bar{PG}}_2(i)$$. Note that for $${\bar{PG}}_2(i)$$, $$x_v=0$$ for any *v* who belongs to any face $$f\equiv i$$ or $$i+1$$$$(mod\, k)$$. $${\bar{PG}}_2(i)$$ consists of independent $$k'$$-outerplanar graphs, each of which has some boundary condition i.e. the emission amount of any vertex in any first and last face is zero and $$k'\le k$$. Suppose the number of these independent $$k'$$-outerplanar graphs is $$L^i$$. Without loss of generality, suppose these $$k'$$-outerplanar graphs are ordered from exterior to interior as $$G_s=(V_s,E_s)$$, $$s\in [L^i]$$ (e.g. $$G_s$$ is the subgraph of *G* constructed by all the vertices of levels from $$(s-2)k+i+1$$ to $$(s-1)k+i$$, $$s=2,\ldots ,L^i-1$$, with boundary $$x_v=0$$ if *v* is of level $$(s-2)k+i+1$$ or $$(s-1)k+i$$, see Fig. 8).

Let $\varPsi _s(Q^s)$ denote the optimal value if there is a solution such that the total allocated scaled emission amount to $G_s$ is exactly $Q^s$ with boundary condition and $\varPsi _s(Q^s)=0$ otherwise, which can be solved by the above DP approach on $k'$-outerplanar graphs with boundary conditions. Then, it is not difficult to see the optimal solution for ${\bar{PG}}_2(i)$ is the optimal solution of the following integer linear program (denoted by *SUB*):Let $g_t(Q)$ denote the optimal integer value of *SUB* when only $G_s$, $s\in [t]$ is considered and the total emission amount allocated to these graphs is exactly *Q*. Then we have the following recursion function (which is essentially the same as that in Lemma 1):

$$\begin{aligned} g_t(Q)=\max _{Q^t=0,1,\ldots , Q}\{g_{t-1}(Q-Q^t)+\varPsi _t(Q^t)\} \end{aligned}$$

The optimal value of *SUB* is $\max _{Q=0,1,\ldots ,p}\{g_{L^i}(Q)\}$, which gives the optimal solution of ${\bar{PG}}_2(i)$ by tracking the optimal value of this dynamic programming approach. The running time of this approach is $O(|L^i|p^2)$. Hence, the total running time for obtaining and solving ${\bar{PG}}_2(i)$ is

$$\begin{aligned} O\left( |L^i|p^2\right) +\sum _{s\in [L^i]}O\left( k|V_s|^3q^{6k+2} \left\lceil \frac{2\rho }{\delta }\right\rceil ^{6k}\right) =O\left( k|V|^3q^{6k+2} \left\lceil \frac{2\rho }{\delta }\right\rceil ^{6k}\right) \end{aligned}$$

We need to solve ${\bar{PG}}_2(i)$, for each $i\in [k]$ and then get $\max _{i\in [k]}\{OPT({\bar{PG}}_2(i))\}$. Therefore, the overall running time is $O(k^2|V|^3q^{6k+2} \lceil \frac{2\rho }{\delta }\rceil ^{6k})$ as desired. $\square$

Let $\frac{2}{k}=\epsilon$ in Theorem 13. Also note that $\rho =|V|(\lfloor \log _2(q)\rfloor +2)$. We have:

#### Theorem 14

For fixed $\delta ,\epsilon >0$ there is an

14 $$\begin{aligned} O\left( \frac{1}{\epsilon ^2}|V|^{12/\epsilon +3}q^2\left\lceil \frac{2(\lfloor \log _2q\rfloor +2)q}{\delta }\right\rceil ^{12/\epsilon +1}\right) =\left( \frac{|V|q(\log _2q+2)}{\delta }\right) ^{O\left( \frac{1}{\epsilon }\right) } \end{aligned}$$

time algorithm for PG on directed planar graph $G=(V,E)$ that achieves social welfare $(1-\epsilon )OPT^{in}(PG)$ and violates each local constraint by a factor of $1+\delta$. This is a PTAS for PG with polynomial size integer variables.

## General Objective Function for Bounded Degree Graphs

### Approximation Algorithms

If *R*(*x*) is monotone, we present an algorithm with an approximation ratio of $O(\Delta )$ for PG on a graph with maximum degree $\Delta$.

#### Theorem 15

If *R*(*x*) with binary variables is monotone increasing, then there is an $(\rho ^{fr}=\frac{e\gamma _{\Delta +2}}{e-1}+1)$-approximation algorithm for PG with integer variables.

#### Proof

If $x_v\in \{0,1\}, \, \forall v\in V$, for any $A\subseteq V$, we define $g(A)=R(x)$ where $x_v=1$, $\forall v\in A$ and $x_v=0$, for any $v\notin A$. It is not difficult to see that *R* with binary variables is submodular if and only if *g* satisfies $g(A\cup B)+g(A\cap B)\le g(A)+g(B)$, for any $A, B \subset V$. For any $A\subseteq V$, and $v\in V$, denote by $A+v$ the set $A\cup \{v\}$. Let $g_v(A)=g(A+v)-g(A)$. Then it is not difficult to see that $g(A\cup B)+g(A\cap B)\le g(A)+g(B)$, for any $A, B \subset V$ if and only if for any $A\subseteq B\subseteq V$ and $v\in V\backslash B$, $g_v(A)\ge g_v(B)$. Next we will prove that $g_v(A)\ge g_v(B)$, which implies that *R*(*x*) is submodular.

Let $A\subseteq B\subseteq V$ and $v\in V\backslash B$. Denote by $\Delta r^{A+v}_u$ the total welfare change of player *u* by adding *v* to set *A*. Observe that

$$\begin{aligned} g_v(A)=\Delta r^{A+v}_v+\sum _{u\in \delta ^{-}_G(v)\cap A}\Delta r^{A+v}_u. \end{aligned}$$

By simple calculations,

$$\begin{aligned} \Delta r^{A+v}_v= & {} b_v\left( 1\right) -b_v\left( 0\right) -d_v\left( 1+\sum _{u\in \delta ^{-}_G\left( v\right) \cap A}w_{uv}x_u\right) +d_v\left( \sum _{u\in \delta ^{-}_G\left( v\right) \cap A}w_{uv}x_u\right) ,\\ \Delta r^{A+v}_u= & {} \,-d_u\left( \sum _{u'\in \delta ^{-}_G\left( u\right) \cap A}\left( w_{u'u}x_{u'}+w_{vu}\right) \right) +d_u\left( \sum _{u'\in \delta ^{-}_G\left( u\right) \cap A}w_{u'u}x_{u'}\right) . \end{aligned}$$

By convexity of $d_u$, we know that

15 $$\begin{aligned} \begin{aligned} \Delta r^{A+v}_v&\ge \Delta r^{B+v}_v, \forall u \in \delta _G(v)\cap A\\ \Delta r^{A+v}_u&\ge \Delta r^{B+v}_u, \forall u \in \delta _G(v)\cap A\\ \Delta r^{B+v}_u&\ge 0, \forall u \in \delta ^{-}_G(v)\cap B \end{aligned} \end{aligned}$$

Hence, $g_v(A)\ge g_v(B)$ and *R*(*x*) with binary variables is submodular.

For the graph with degree $\Delta$, note that PG is $\Delta +2$ column sparse. By Proposition 2, there is a randomized $\rho ^{fr}=\frac{e\gamma _{\Delta +2}}{e-1}$-approximate algorithm for PG with binary variables if *R*(*x*) is monotone increasing, because such *R*(*x*) is also submodular. This algorithm can be derandomized to be deterministic with the same approximation ratio. Now observe that for a concave function *G*(*x*) from $\mathbb {R}_{\ge 0}$ to $\mathbb {R}_{\ge 0}$, we have $G(x+y)\le G(x)+G(y)$, for any *x*, $y\in \mathbb {R}_{\ge 0}$ (without loss of generality let $x\ge y>0$, by concavity of *G*, it holds that $\frac{G(x+y)-G(x)}{x+y-x}\le G'(x)\le G'(y)\le \frac{G(y)-G(0)}{y-0}$). By this property, since $b_v(x)$ and $-d_v(x)$ are concave from $\mathbb {R}_{\ge 0}$ to $\mathbb {R}_{\ge 0}$, for any $v\in V$, for any feasible solution $x=\{x_v\}_v$ and $y=\{y_v\}_v$, we have $R(x+y)\le R(x)+R(y)$. By ellipsoid algorithm for convex programming problem in [25], we can get an optimal fractional solution of PG denoted as $x^*=\{x^*_v\}_v$. Let $z^*=\{z^*_v\}_v$ where $z^*_v=\lfloor x^*_v\rfloor$, for any $v\in V$ and $x^*=z^*+y^*$. Let $y'$ be an $\frac{e\gamma _{\Delta +2}}{e-1}$-approximate solution for PG with binary variables when *R*(*x*) is monotone increasing. Note that $y^*$ is a feasible fractional solution for PG with binary variables. We know $R(y^*)\le \frac{e\gamma _{\Delta +2}R(y')}{e-1}$. Let $x'$ be an solution of PG with integer variables such that $x'=y'$ if $R(y')\ge R(z^*)$ and $x'=z^*$ otherwise. Therefore, we have $R(x^*)\le R(z^*)+R(y^*)\le R(z^*)+\frac{e\gamma _{\Delta +2}R(y')}{e-1}\le (\frac{e\gamma _{\Delta +2}}{e-1}+1)R(x')$. $\square$

### Truthful in Expectation Mechanisms

**Fig. 9:**
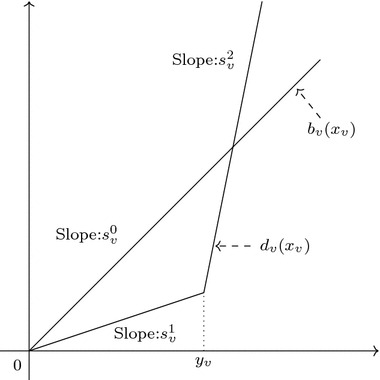
An illustration of function $b_v$ and $d_v$

In this section, we will prove that there is an $O(\Delta +2)$ truthful in expectation mechanism for PG on any graph with degree $\Delta$ when $b_v$ is linear and $d_v$ is piece-wise linear with one shift point, and a little further natural assumption. For each player $v\in V$, let $b_v(x_v)=s^0_vx_v$, a linear function starting from the origin with slope $s^0_v$. $d_v$ is a piece-wise linear convex function with one shift point where the first part is a linear function starting from the origin with slope $s^1_v$, the shift point is $(y_v, s^1_v y_v)$ and the second part is a linear function starting from the shift point with slope $s^2_v\ge s^1_v$ (see Fig. 9).

As we know if the emitted pollution from player *v* is large enough, the welfare of player *v* should be negative. The damage function is piece-wise linear with one shift point, which means player *v*’s welfare (valuation minus damage) will decrease after the total pollution in *v* reaches $y_v$. Precisely, when $x_v+\sum _{u\in \delta ^{-}_G(v)} w_{uv}x_u\ge y_v$, we should have $s^0_v\le s^2_v$. However, we can relax this condition to

16 $$\begin{aligned} s^0_v- s^2_v-\sum _{u\in \delta _G^{+}(v)}s^1_uw_{vu}\le 0. \end{aligned}$$

The second condition on the slope $s^0_v$ is somehow more subtle. Intuitively, player *v*’s emitted pollution should not affect his neighbour’s total pollution too much. This means that if his neighbour *u*’s pollution reaches $y_u$, then the total social welfare *R*(*x*) should decrease. That is, for any $v\in V$, and any $u\in \delta _G^{+}(v)$, if $\sum _{u'\in \delta _G^{-}(u)}w_{u'u}x_{u'}\ge y_u$, then

17 $$\begin{aligned} s^0_v- s^1_v-\sum _{u'\in \delta ^{+}_G(v)\backslash \{u\}}s^1_{u'}w_{vu'}\le s^2_uw_{vu}. \end{aligned}$$

#### Lemma 10

Let $x^*$ be an optimal fractional solution of PG under the condition that functions $b_v$ and $d_v$ satisfy constraints (16) and (17), then $x^*$ can have the following property: for each $v\in V$, the local level of pollution in *v* satisfies that $x^*_v+\sum _{u\in \delta ^{-}_G(v)} w_{u v}x^*_u\le y_v$.

#### Proof

We prove this lemma by contradiction. Suppose there exists $v \in V$, such that $x^*_v+\sum _{u\in \delta ^{-}_G(v)} w_{u v}x^*_u> y_v$. If $x^*_v>0$, by constraints (16), we can decrease the value of $x^*_v$ by an amount of $\alpha$ such that $x^*_v-\alpha +\sum _{u\in \delta ^{-}_G(v)} w_{u v}x^*_u> y_v$. By simple calculation, the total social welfare increases by an amount of $-(s^0_v- s^2_v-\sum _{u\in \delta ^{+}_G(v)}s^1_uw_{vu})\alpha \ge 0$. Thus, we can do this until either $x^*_v=0$ or $x^*_v-\alpha +\sum _{u\in \delta ^{-}_G(v)} w_{u v}x^*_u\le y_v$. If the first case holds and the second case does not hold, then there exists $u\in \delta ^{-}_G(v)$ with $x^*_u>0$. Note that $v\in \delta ^{+}_G(u)$. Since $\sum _{u\in \delta ^{-}_G(v)} w_{u v}x^*_u> y_v$, if we decrease the value $x^*_u$ by $\alpha$, by simple calculation, the total social welfare increases by at least $-(s^0_u- s^{i}_u-\sum _{u'\in \delta ^{+}_G(u)\backslash \{v\}}s^{i}_{u'}w_{uu'}- s^2_vw_{uv})\alpha \ge -(s^0_u- s^{1}_u-\sum _{u'\in \delta ^{+}_G(u)\backslash \{v\}}s^{1}_{u'}w_{uu'}- s^2_vw_{uv})\alpha$, which is non-negative by constraints (17). Here, the value *i* is defined as follows, if total pollution in *v* is below $y_u$ then $s^{i}_u=s^{1}_u$, otherwise $s^{i}_u=s^{2}_u$, with the same argument for $s^{i}_{u'}$. By this operation, we can decrease the value of *v* without loss of total social welfare until the total level of pollution in *v* does not reach $y_v$. $\square$

#### Lemma 11

If PG functions $b_v$ and $d_v$ satisfy constraints (16) and (17) then there is a deterministic polynomial time algorithm with approximation ratio $\rho ^{fr}=\gamma _{\Delta +2}$.

#### Proof

By Lemma 10, we know the optimal fractional solution $x^*$ can satisfy that $x^*_v+\sum _{u\in \delta ^{-}_G(v)} w_{u v}x^*_u\le y_v$, for any $v\in V$. Hence, we can modify the constraint (7) in PG to 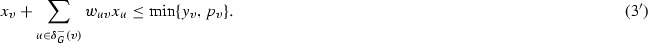


This modified PG has the same optimal fractional solution as PG. In the modified PG, $R(x)=\sum _{v\in V}\omega _vx_v$, where $\omega _v=s^0_v-s^1_v-\sum _{u\in \delta ^{-}_G(v)}s^1_uw_{vu}$. By Proposition 1, there is a deterministic polynomial time algorithm for the modified PG with approximation ratio $\rho ^{fr}=\gamma _{\Delta +2}$. This algorithm is also an algorithm for PG with the same approximation ratio. $\square$

With Lemma 11, we now present a truthful in expectation mechanism for PG with approximation ratio $\gamma _{\Delta +2}=(e+o(1))(\Delta +2)$.

#### Theorem 16

Suppose the bidding strategy $s^0_v$ of each player $v \in V$ satisfies constraints (16) and (17). There is a randomized, individually rational, $(\rho ^{fr}=\gamma _{\Delta +2})$-approximation mechanism that is truthful in expectation for PG on *G* with degree at most $\Delta$.

#### Proof

Since the bidding strategy $s^0_v$ of each player *v* satisfies constraints (16) and (17), by Proposition 3 and Lemma 11, there is a randomized, individually rational, $\gamma _{\Delta +2}$-approximation mechanism that is truthful in expectation for the modified PG, which is also a truthful in expectation mechanism for PG with the same approximation ratio. $\square$

#### Corollary 2

If $b_v$ and $d_v$ are linear functions for any *v* and the bidding strategy $s^0_v$ of player *v* is arbitrary, then there is a randomized, individually rational, $(\rho ^{fr}=\gamma _{\Delta +2})$-approximation mechanism that is truthful in expectation for PG.

#### Proof

Since $d_v$ is linear, it is equivalent to the above piece-wise linear function with $s^2_v=+\infty$ and $y_v=+\infty$, for any $v\in V$. By Theorem 16, there is a randomized, individually rational, $\gamma _{\Delta +2}$-approximation mechanism that is truthful in expectation for PG. $\square$

#### Remark

We cannot anticipate an algorithm with constant approximation ratio for PG on the graph with average degree $\Delta$ (the average degree of a graph *G* is $\frac{\sum _{v\in V}|\delta _{G^{un}}(v)|}{|V|}$) even if $\Delta =1$. Consider a graph $G'$ consisting of a complete graph with *n* vertices and $n^2-n$ isolated vertices with valuation 0. Note that $G'$’s average degree is $\frac{n^2}{n^2}= 1$. PG on *G* cannot be approximated within $n^{1-\epsilon }$ unless Unique Game conjecture fails. Thus, PG cannot be approximated within $(n^2)^{\frac{1-\epsilon }{2}}$ on $G'$, where $n^2$ is the number of vertices of $G'$.

## Open Problems

We presented a new network model for the pollution control problem and studied planar and tree networks which model realistic scenarios. These networks can be applied to model air and water pollution from diffuse sources. Our main technical results include a constant approximation algorithm and a PTAS with a small violation in the constraints for the case of planar graphs and an FPTAS which is truthful in expectation and a 3-approximate deterministic truthful mechanism for the case of trees. We obtained these results by introducing novel algorithmic techniques for planar and tree graphs which could be of independent interest.

Many interesting open problems arise from this new model. Our main open question is to determine whether PG with binary variables on planar graphs admits a PTAS or whether it is APX-hard, when no local constraint is volated. Another direction would be to study lower bounds on truthful (deterministic, universal, truthful in expectation) mechanisms for PG. Can externality be used to obtain such lower bounds? Furthermore it would be interesting to generalize our results to other graphs, e.g., Euclidean graphs.

## Footnotes

1 Nonpoint source (NPS) pollution refers to both water and air pollution from diffuse sources, that is sources without a specified fixed location. For instance, nonpoint source water pollution affects a water body from sources such as polluted runoff from agricultural areas draining into a river, or wind-borne debris blowing out to sea. This work deals mainly with point source pollution.

2 The author of [38] uses cost function rather than benefit function, which can be viewed as $M_v-b_v(x_v)$, with $M_v$ a large constant for any $v\in V$. The author assumes that cost function is convex decreasing and it is equivalent to $b_v(x_v)$ being a concave increasing function. We use benefit function rather than cost function for ease of analysis.

3 Fully Polynomial Time Approximation Scheme, Polynomial Time Approximation Scheme and Efficient Polynomial Time Approximation Scheme respectively.

4 This PTAS also works for multiple choice and constant dimensional knapsack problem, which will be used for PG with polynomial size integer variables.
